# Supplementary material for: Leveraging eQTLs to identify individual-level tissue of interest for a complex trait
Source: PLoS Comput Biol. 2021 May 21;17(5):e1008915. doi: 10.1371/journal.pcbi.1008915 (PMC8174686; doi:10.1371/journal.pcbi.1008915)
Supplement: S1 Text — (PDF) [file pcbi.1008915.s001.pdf]

# S1 Text: Markov Chain Monte Carlo (MCMC) algorithm for eGST

Our model for the phenotype of individual $i$ is:

$$\begin{aligned} y_i &= \alpha_1 + \boldsymbol{x}_{1i}^{'}\boldsymbol{\beta}_1 + \epsilon_{1i} \text{ if } C_i = 1 \\ &= \alpha_2 + \boldsymbol{x}_{2i}^{'}\boldsymbol{\beta}_2 + \epsilon_{2i} \text{ if } C_i = 2 \end{aligned} \tag{6}$$

Here, $\alpha_k$ is the baseline tissue-specific trait mean, $\boldsymbol{x}_{ki}$ is the vector of normalized genotype values of individual $i$ at the eQTL SNPs specific to tissue $k$, $\boldsymbol{\beta}_k = (\beta_{k1}, \beta_{k2} \ldots, \beta_{km_k})$ are their effects on the trait under $C_i = k$, and $\epsilon_{ki}$ is a noise term, $i = 1, \ldots, n$ and $k = 1, 2$. The random errors are distributed as: $\epsilon_{1i} \sim \mathrm{N}(0, \sigma^2_{\epsilon_1})$ and $\epsilon_{2i} \sim \mathrm{N}(0, \sigma^2_{\epsilon_2})$.

Given that $i = 1$, we note that: $V(\beta_{1j}) = \sigma^2_{x_1}$ and $V(\boldsymbol{x}_{11}^{'}\boldsymbol{\beta}_1) = m_1\sigma^2_{x_1}; V(\beta_{2j}) = \sigma^2_{x_2}$ and $V(\boldsymbol{x}_{21}^{'}\boldsymbol{\beta}_2) = m_2\sigma^2_{x_2}$. We define $\tau_1$ and $\tau_2$ such that: $V(\boldsymbol{x}_{11}^{'}\boldsymbol{\beta}_1) = \tau_1 V(\epsilon_{11}) = \tau_1\sigma^2_{\epsilon_1}$ and $V(\boldsymbol{x}_{21}^{'}\boldsymbol{\beta}_2) = \tau_2 V(\epsilon_{21}) = \tau_2\sigma^2_{\epsilon_2}$. Thus, under $C = 1$, total variance of the trait is: $V(\boldsymbol{x}_{11}^{'}\boldsymbol{\beta}_1) + V(\epsilon_{11}) = m_1\sigma^2_{x_1} + \sigma^2_{\epsilon_1} = \tau_1\sigma^2_{\epsilon_1} + \sigma^2_{\epsilon_1} = (\tau_1 + 1)\sigma^2_{\epsilon_1}$. Hence, when $C = 1$, the heritability of the trait is: $\frac{\tau_1\sigma^2_{\epsilon_1}}{(\tau_1+1)\sigma^2_{\epsilon_1}} = \frac{\tau_1}{\tau_1+1}$. So, $k^{th}$ tissue-specific subtype heritability of the trait is $\frac{\tau_k}{\tau_k+1}$. Since, specifying $\sigma^2_{\epsilon_k}$ and $\tau_k$ determine $\sigma^2_{x_k} (= \frac{\tau_k\sigma^2_{\epsilon_k}}{m_k})$, we place prior distributions on $\sigma^2_{\epsilon_k}$ and $\tau_k$ and update these parameters in the MCMC. From the MCMC sample of $\tau_k$, we can estimate the $k^{th}$ tissue-specific subtype heritability of the trait. The prior distributions of the $k^{th}$ tissue-specific set of parameters are given by: for $k = 1, 2$,

$$\begin{gathered} \alpha_k \sim N(0, \sigma^2_\alpha);\ \boldsymbol{\beta}_k \sim N(\mathbf{0}, \sigma^2_{x_k} I_{m_k});\ \sigma^2_{\epsilon_k} \sim \text{Inverse-Gamma}(a_\epsilon, b_\epsilon); \\ \tau_k \sim \text{Beta}(\psi, 1);\ P(C_i = k | w_1, w_2) = w_k;\ (w_1, w_2) \sim \text{Beta}(s_1, s_2). \end{gathered}$$

For ease in presentation of the MCMC algorithm, we define the following terms:

1. $m_k$ = number of $k^{th}$ tissue-specific eQTLs.
2. $n_k = \#\{i : C_i = k\}$ = number of individuals assigned to $k^{th}$ tissue-specific subtype.
3. $\sigma^2_{\alpha_k} = \frac{1}{\frac{n_k}{\sigma^2_{\epsilon_k}} + \frac{1}{\sigma^2_\alpha}}$.
4. $G_k$ is a $(m_k \times n_k)$ matrix which is a sub-matrix of $X_k$ of order $(m_k \times n)$, the columns of $G_k$ are selected from the columns of $X_k$ corresponding to the individuals such that $C_i = k$. Of note, $G_k$ and $X_k$ both have $m_k$ rows. $\boldsymbol{y}_k = \{y_i - \alpha_k : C_i = k\}$, i.e., it is a sub-vector of $(\boldsymbol{y} - \alpha_k)$ corresponding to the individuals such that $C_i = k$. So, the sub-vector has length $n_k$.

# MCMC algorithm for eGST

S1 Algorithm: MCMC algorithm based on Gibbs sampling to implement eGST

1: **Initialization**: First we run the MAP-EM algorithm for 15 iterations to obtain an initial values of the various parameters to be updated in each MCMC step. We initialize $\sigma^2_{x_k}, k = 1, 2$, as the sample variance of $\boldsymbol{\beta}_k$ values obtained from the last iteration of the MAP-EM algorithm, and initialize $\tau_k$ accordingly. Next, we update the different parameters in each MCMC step.

2: **Loop**: For each $k = 1, 2$:

3: Update $\alpha_k$ which has the full conditional posterior distribution $N(\mu_{\alpha_k}, \sigma^2_{\alpha_k})$ with $\mu_{\alpha_k} = \frac{\sigma^2_{\alpha}}{\sigma^2_{\epsilon_k}} \sum_{i:C_i=0} z_{ik}$; where $z_{ik} = y_i - \boldsymbol{x}'_{ki}\boldsymbol{\beta}_k$.

4: Update $\boldsymbol{\beta}_k$ from the following: $N_{m_k}(\boldsymbol{\mu}_k, S_k)$, $S_k = \left(\frac{1}{\sigma^2_{\epsilon_k}} G_k G'_k + \frac{1}{\sigma^2_{x_k}} I_{m_k}\right)^{-1}$, $\boldsymbol{\mu}_k = \frac{1}{\sigma^2_{\epsilon_k}} S_k G_k \boldsymbol{y}_k$.

5: Separately update each $C_i, i = 1, \ldots, n$, with $1^{st}$ tissue-specific posterior probability $q_{i1} = \frac{1}{1+\text{ratio}_{ik}}$, where $\text{ratio}_{ik} = \frac{w_2}{w_1} \frac{\sigma_{\epsilon_1}}{\sigma_{\epsilon_2}} exp[\frac{1}{2}(comp_{i1} - comp_{i2})]$, where $comp_{i1} = \frac{1}{\sigma^2_{\epsilon_1}}(y_i - \alpha_1 - \boldsymbol{x}'_{1i}\boldsymbol{\beta}_1)^2$ and $comp_{i2} = \frac{1}{\sigma^2_{\epsilon_2}}(y_i - \alpha_2 - \boldsymbol{x}'_{2i}\boldsymbol{\beta}_2)^2$. We note that: $q_{i2} = 1 - q_{i1}$.

6: Update $w_1$ as: $w_1 \sim \text{Beta}(n_1 + s_1, n_2 + s_2)$. We note that: $w_2 = 1 - w_1$.

7: Update $\sigma^2_{\epsilon_k}$ from Inv-Gamma$(\frac{n_k}{2} + \frac{m_k}{2} + a_\epsilon, T_{1k} + T_{2k} + b_\epsilon)$; where $T_{1k} = \frac{1}{2} \sum_{i:C_i=k}(y_i - \alpha_k - \boldsymbol{x}'_{ki}\boldsymbol{\beta}_k)^2$, and $T_{2k} = \frac{m_k \boldsymbol{\beta}'_k \boldsymbol{\beta}_k}{2\tau_k}$, $k = 1, 2$.

8: $\tau_k \sim$ truncated Inv-Gamma$(\frac{m_k}{2} - \psi, CT_k)$, $0 < \tau_k < 1$, $CT_k = \frac{m_k}{2\sigma^2_{\epsilon_k}} \boldsymbol{\beta}'_k \boldsymbol{\beta}_k$

9: Compute $\sigma^2_{x_k} = \frac{\tau_k \sigma^2_{\epsilon_k}}{m_k}$, and $k^{th}$ tissue-specific subtype heritability $= \frac{\tau_k}{\tau_k + 1}$.

10: Go back to step 2 until all the MCMC iterations are completed.
