# Supplementary material for: Leveraging eQTLs to identify individual-level tissue of interest for a complex trait
Source: PLoS Comput Biol. 2021 May 21;17(5):e1008915. doi: 10.1371/journal.pcbi.1008915 (PMC8174686; doi:10.1371/journal.pcbi.1008915)
Supplement: S2 Text — (PDF) [file pcbi.1008915.s002.pdf]

S2 Text: phenotypic characteristics of AS and MS tissue-specific groups of individuals for WHRadjBMI

We explored the phenotypic characteristics of the individuals assigned with a prioritized tissue for WHRadjBMI [11,803 AS (adipose subcutaneous) specific subtype individuals and 7,238 MS (muscle skeletal) specific subtype individuals (S1 Table)]. We considered 106 phenotypes in the UK Biobank and tested each one for being differentially distributed (heterogeneous) between individuals of each tissue-specific subtype and the remaining population (see Methods). In aggregate, 37 quantitative traits and 37 qualitative traits (total 74 among 106) were significantly heterogeneous between at least one of the WHRadjBMI-AS or WHRadjBMI-MS specific groups versus the remaining population. For example, various *blood counts*, *body size measures*, *medical conditions, urine assays, townsend deprivation index, age at which full time education completed* (S11 Table), *sex (male/female), diabetes proportion*, various mental health phenotypes (S13 Table), *alcohol intake frequency, sleeplessness insomnia* (S14 Table), etc., were differentially distributed between one or both tissue-specific subtype groups of individuals versus the remaining population. But, none of these 106 traits was found to be differentially distributed between a random set of individuals from the population (with the same size as a tissue-specific subtype group) and the remaining population (see Methods).

23 quantitative and 28 categorical traits showed heterogeneity in both the AS group versus the population, and the MS group versus population. We found 6 quantitative and 5 qualitative traits heterogeneous for individuals in the AS group but not the MS group, and found 8 quantitative and 4 qualitative traits heterogeneous for individuals in the MS group but not in the AS group (S11, S13 and S14 Tables). We can interpret the following finding as subtype specificity: while *townsend deprivation index at recruitment* and *neuroticism score* were heterogeneous for both AS and MS tissue-specific subtypes; *creatinine enzymatic in urine* was heterogeneous only for AS and *basophil count* only for MS (S11 Table). Similarly, *sex* (male/female) and *diabetes diagnosed by doctor* were heterogeneous for both tissue-specific subtypes but *risk taking* was heterogeneous only for AS and *pregnant* was heterogeneous for MS (S13 Table).

For a majority of the quantitative traits (except *reticulocyte count, monocyte count, non cancer illness code self reported*), the relative difference of the trait between individuals of a tissue-specific subtype and the remaining population (tissue-specific relative change, see Methods) are in the same direction across tissues (S5 Fig). Among binary traits, *ever smoked* was more prevalent in MS-specific group and less prevalent in AS-specific group when compared to the population (S6 Fig). We observe that for most of the case-control

traits, both the tissue-specific groups of individuals had a higher risk of developing the disease compared to the population (S6 Fig). When the tissue-specific relative change of the traits (see Methods) were in the same direction across tissues, they were of different magnitude for a majority of the traits (S5 and S6 Figs).

Since WHRadjBMI itself was differentially distributed between the individuals of AS (as well as MS) specific subtype compared to the remaining population, we investigated whether the heterogeneity of 72 non-WHR traits (S11, S13 and S14 Tables) were induced due to WHRadjBMI heterogeneity (see Methods). After WHRadjBMI adjustment, 32 quantitative traits (S5 Fig) among 37 primarily heterogeneous quantitative traits (S11 Table) remained heterogeneous between at least one of AS and MS specific groups and the remaining population. Similarly, out of 37 qualitative traits, 27 traits (S6 Fig), S14 Table) remained heterogeneous for at least one of the tissue specific groups after WHRadjBMI adjustment. These results are consistent with unique phenotypic characteristics of these individuals beyond the main phenotype effect. The WHRadjBMI-adjusted tissue-specific relative change of a quantitative trait compared to the population was computed based on the trait residuals (obtained after WHRadjBMI adjustment in the population) instead of the trait itself (see Methods). For some phenotypes, WHRadjBMI-adjusted tissue-specific relative change was in opposite direction across tissues, but in the same direction for other phenotypes (S5 Fig). Since we used linear regression while evaluating WHRadjBMI-adjusted tissue-specific relative change of heterogeneous non-WHR quantitative traits (S5 Fig, S16 Table), we also investigated a model-free WHRadjBMI random matching strategy. We assessed the magnitude of relative change of a trait between individuals with the AS (or MS) subtype and a group of WHRadjBMI-matched random individuals drawn from the population (see Methods). For example, the magnitude of primary AS-specific relative change (prior to WHRadjBMI matching) for *sitting height* and *standing height* decreased from 16% and 22% to 4% and 3% after WHRadjBMI matching, respectively (S18 Table).
