# Supplementary material for: Leveraging eQTLs to identify individual-level tissue of interest for a complex trait
Source: PLoS Comput Biol. 2021 May 21;17(5):e1008915. doi: 10.1371/journal.pcbi.1008915 (PMC8174686; doi:10.1371/journal.pcbi.1008915)
Supplement: S1 Fig — (PDF) [file pcbi.1008915.s003.pdf]

a

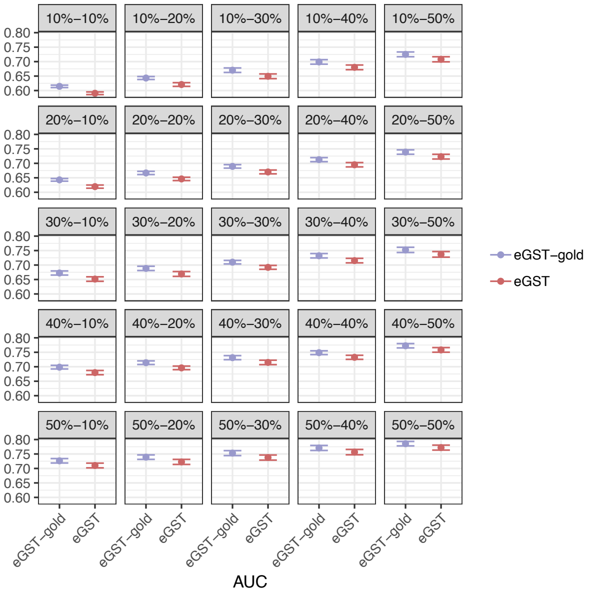

b

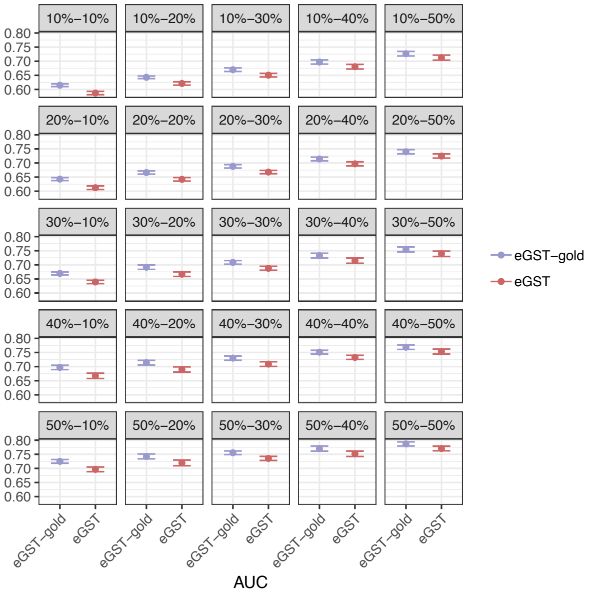

S1 Fig: Box plots of AUCs comparing the classification accuracy of eGST and the gold-standard strategy implementing our model under the following simulation scenarios: $\boldsymbol{m_1 = m_2 = 1000}$, all possible combinations of (h_1^2, h_2^2) with $h_1^2 = 10\%, 20\%, 30\%, 40\%, 50\%$ and $h_2^2 = 10\%, 20\%, 30\%, 40\%, 50\%$; $n = 40000$; (**a**) $w_1 = w_2 = \frac{1}{2}$ and (**b**) $w_1 = \frac{1}{3}, w_2 = \frac{2}{3}$. Here h_1^2 and h_2^2 are the heritabilities of tissue-specific subtypes of the trait due to m_1 and m_2 SNPs representing two sets of tissue-specific eQTL SNPs, w_1 and w_2 are the proportions of individuals in the sample assigned to the two tissues, n is the total number of individuals. The box plots of AUC were constructed based on 50 datasets simulated under each scenario. In the gold standard strategy, true model parameters were assumed to be known while estimating the tissue-specific posterior probabilities.
