# Supplementary material for: Leveraging eQTLs to identify individual-level tissue of interest for a complex trait
Source: PLoS Comput Biol. 2021 May 21;17(5):e1008915. doi: 10.1371/journal.pcbi.1008915 (PMC8174686; doi:10.1371/journal.pcbi.1008915)
Supplement: S4 Fig — (PDF) [file pcbi.1008915.s006.pdf]

a

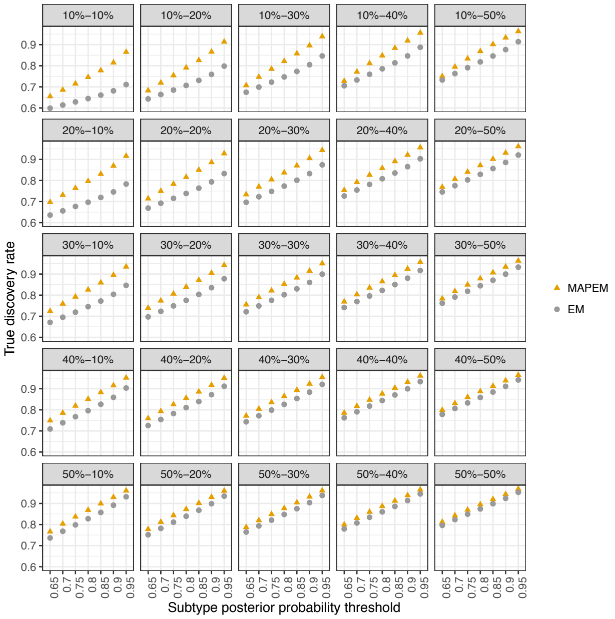

b

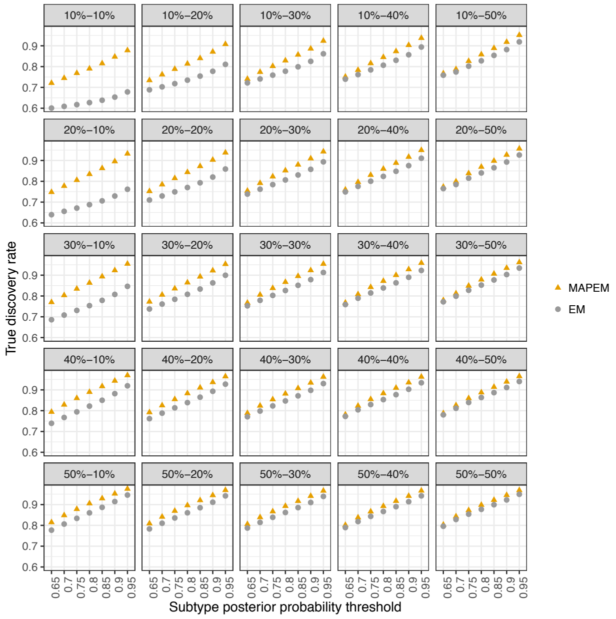

S4 Fig: Comparison between the true discovery rate (TDR) of classifying tissue-specific subtypes by the MAP-EM algorithm (under the Bayesian framework of the mixture model which eGST employs) versus the EM algorithm (under the frequentist framework of the mixture model) based on the threshold of tissue-specific subtype posterior probability as $65\%, 70\%, 75\%, 80\%, 85\%, 90\%, 95\%$, respectively. Box plots of TDR across 50 datasets simulated under $\boldsymbol{m_1 = 1000, m_2 = 1500}$, combinations of (h_1^2, h_2^2) with $h_1^2 = 10\%, 20\%, 30\%, 40\%, 50\%$ and $h_2^2 = 10\%, 20\%, 30\%, 40\%, 50\%$; $n = 40000$; (**a**) $w_1 = w_2 = \frac{1}{2}$ and (**b**) $w_1 = \frac{1}{3}, w_2 = \frac{2}{3}$, are presented. Here h_1^2 and h_2^2 are the heritabilities of tissue-specific subtypes of the trait due to m_1 and m_2 SNPs representing two sets of tissue-specific eQTL SNPs, w_1 and w_2 are the proportions of individuals in the sample assigned to two tissues, n is the total number of individuals.
