# Supplementary material for: Leveraging eQTLs to identify individual-level tissue of interest for a complex trait
Source: PLoS Comput Biol. 2021 May 21;17(5):e1008915. doi: 10.1371/journal.pcbi.1008915 (PMC8174686; doi:10.1371/journal.pcbi.1008915)
Supplement: S5 Fig — (PDF) [file pcbi.1008915.s007.pdf]

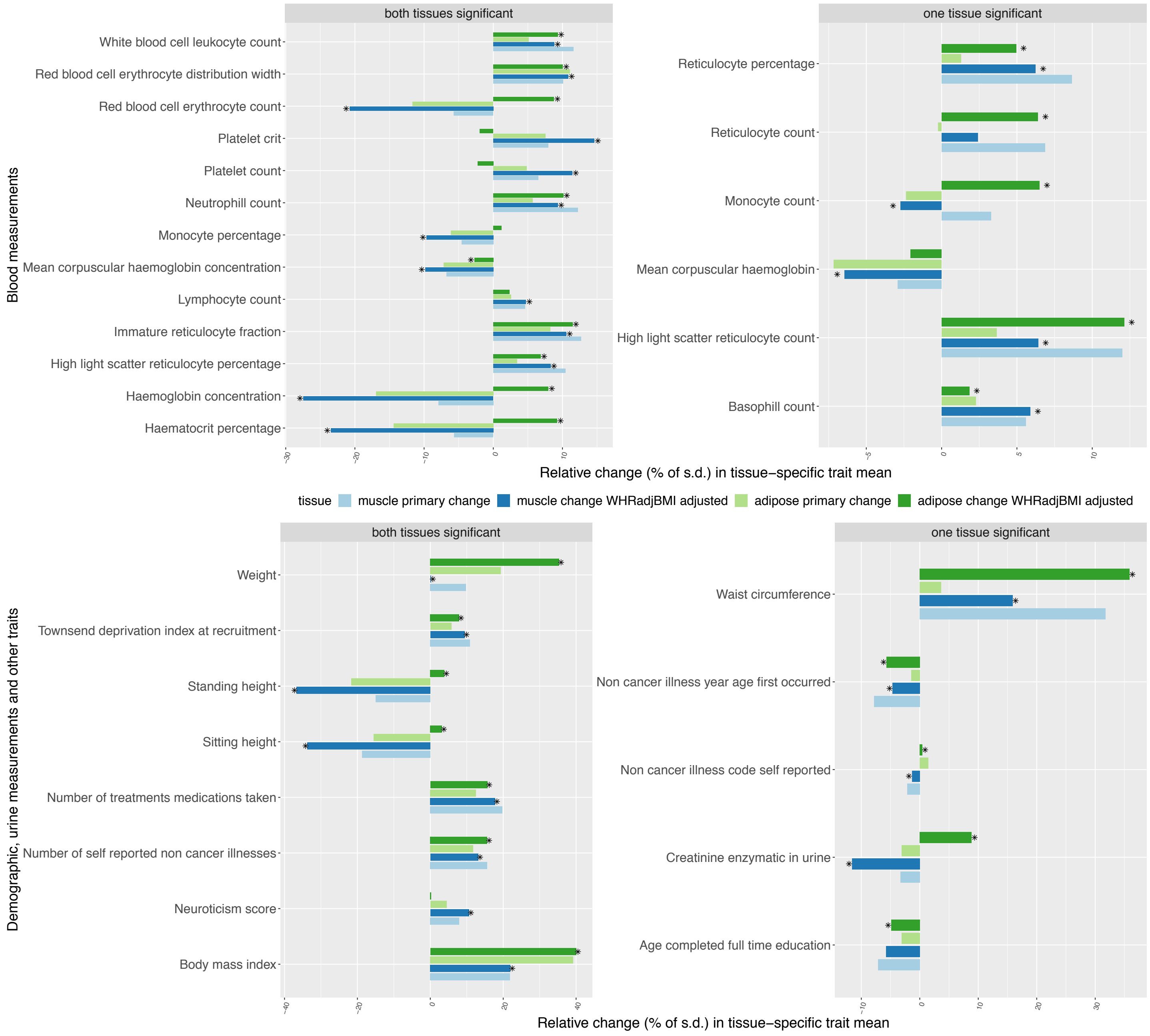

S5 Fig: Percentage of tissue-specific relative change of the quantitative traits differentially distributed between the individuals assigned to a tissue-specific subtype of WHRadjBMI and the remaining population. Traits in the left panels are primarily heterogeneous for both tissue-specific groups and traits in the right panels are heterogenous for one tissue-specific group. We measure the tissue-specific relative change of a trait by: $\frac{\text{tissue specific mean - remaining population mean}}{\text{population s.d.}} \times 100$, where the tissue-specific mean is computed only in the individuals with the corresponding tissue-specific subtype. The same measure is calculated for a trait residual obtained after adjusting for WHRadjBMI to quantify the tissue-specific relative change of the trait after WHRadjBMI adjustment. The faded green (or blue) bar presents primary adipose (or muscle) tissue-specific relative change of a trait compared to the remaining population. The dark green (or blue) bar presents the WHRadjBMI-adjusted adipose (or muscle) specific relative change of a trait. Each trait listed here was found differentially distributed between at least one of adipose or muscle specific group and the remaining population after WHRadjBMI adjustment. For each trait the asterisk mark attached to the bars indicates which tissue-specific group remains significantly heterogeneous after WHRadjBMI adjustment.
