# Supplementary material for: Leveraging eQTLs to identify individual-level tissue of interest for a complex trait
Source: PLoS Comput Biol. 2021 May 21;17(5):e1008915. doi: 10.1371/journal.pcbi.1008915 (PMC8174686; doi:10.1371/journal.pcbi.1008915)
Supplement: S6 Fig — (PDF) [file pcbi.1008915.s008.pdf]

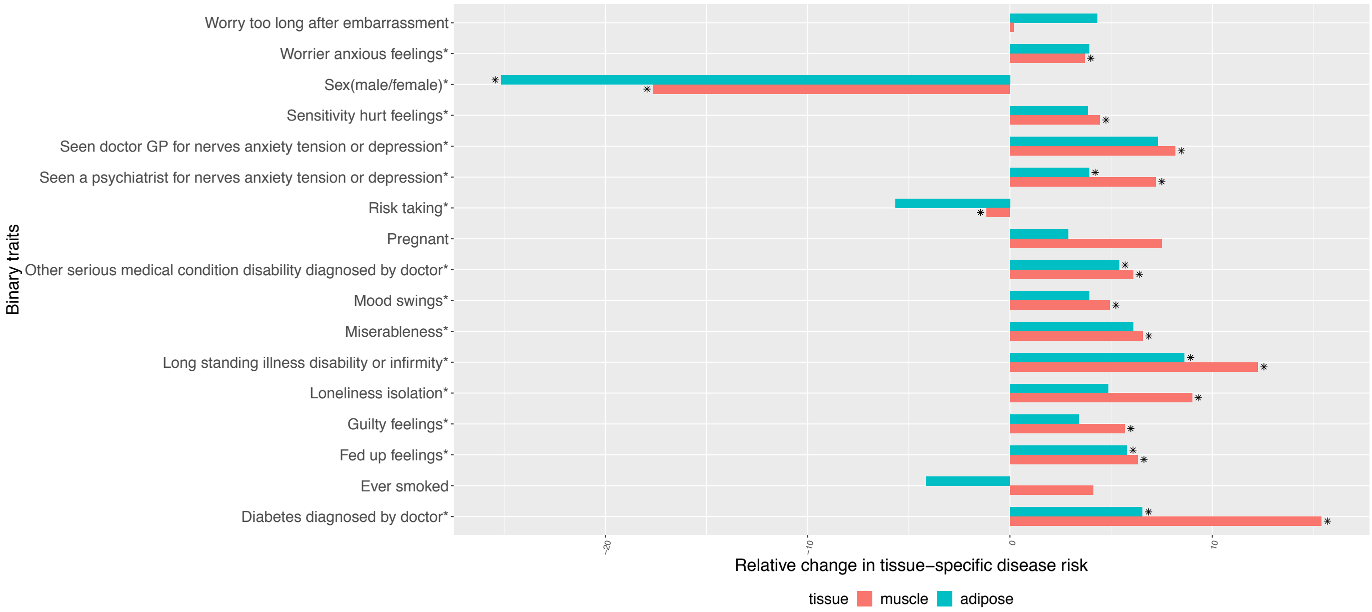

S6 Fig: Percentage of tissue-specific relative change in the risk of case-control traits between the individuals assigned to a tissue-specific subtype of WHRadjBMI and the population. The tissue-specific relative change of a disease risk is measured by: $\frac{\text{tissue specific prevalence - population prevalence}}{\text{population s.d.}} \times 100$. Tissue specific prevalence of the disorder was computed only in the individuals classified as the corresponding tissue-specific subtype of WHRadjBMI. The asterisk mark attached to the traits indicate which trait remains differentially distributed between at least one of adipose and muscle tissue-specific group of individuals and the remaining population after WHRadjBMI adjustment. For each trait the asterisk mark attached to the bars indicate which tissue-specific group of individuals remains significantly heterogeneous for the trait after WHRadjBMI adjustment.
