# Supplementary material for: Leveraging eQTLs to identify individual-level tissue of interest for a complex trait
Source: PLoS Comput Biol. 2021 May 21;17(5):e1008915. doi: 10.1371/journal.pcbi.1008915 (PMC8174686; doi:10.1371/journal.pcbi.1008915)
Supplement: S1 Table — (PDF) [file pcbi.1008915.s009.pdf]

	BMI (total #individuals: 336,106)		WHRadjBMI (total #individuals: 336,018)	
post prob cutoff	Adipose	Brain	Adipose subcutaneous	Muscle skeletal
65%	11,838	13,354	11,803	7,238
70%	5,397	6,465	5,319	2,787

S1 Table: Number of individuals classified by eGST as a tissue-specific subtype of BMI and WHRadjBMI based on tissue-specific posterior probability threshold of 65% and 70%.
