# Supplementary material for: Leveraging eQTLs to identify individual-level tissue of interest for a complex trait
Source: PLoS Comput Biol. 2021 May 21;17(5):e1008915. doi: 10.1371/journal.pcbi.1008915 (PMC8174686; doi:10.1371/journal.pcbi.1008915)
Supplement: S2 Table — (PDF) [file pcbi.1008915.s010.pdf]

simulation scenario	mean AUC	
	$n = 40,000$	$n = 100,000$
$w_1 = w_2 = 0.5, m_1 = m_2 = 1000, h_1^2 = 10\%, h_2^2 = 10\%$	0.59	0.60
$w1 = w2 = 0.5, m_1 = m_2 = 1000, h_1^2 = 20\%, h_2^2 = 20\%$	0.65	0.66

S2 Table: Simulation results: effect of increasing sample size on classification accuracy of eGST.
