# Supplementary material for: Leveraging eQTLs to identify individual-level tissue of interest for a complex trait
Source: PLoS Comput Biol. 2021 May 21;17(5):e1008915. doi: 10.1371/journal.pcbi.1008915 (PMC8174686; doi:10.1371/journal.pcbi.1008915)
Supplement: S3 Table — (PDF) [file pcbi.1008915.s011.pdf]

simulation scenario ($n = 40,000$)	mean AUC	
	$(m_1, m_2) = (1000, 1000)$	$(m_1, m_2) = (1500, 1500)$
$w_1 = w_2 = 0.5, h_1^2 = 10\%, h_2^2 = 10\%$	0.59	0.58
$w_1 = w_2 = 0.5, h_1^2 = 20\%, h_2^2 = 20\%$	0.65	0.64

S3 Table: Simulation results: effect of increasing number of tissue-specific SNPs with fixed subtype heritability per tissue on classification accuracy of eGST.
