# Supplementary material for: Leveraging eQTLs to identify individual-level tissue of interest for a complex trait
Source: PLoS Comput Biol. 2021 May 21;17(5):e1008915. doi: 10.1371/journal.pcbi.1008915 (PMC8174686; doi:10.1371/journal.pcbi.1008915)
Supplement: S4 Table — (PDF) [file pcbi.1008915.s012.pdf]

simulation scenario ($n = 40,000$)	AUC	
	$\alpha_1 = \alpha_2 = 0$	$\alpha_1 = 0, \alpha_2 = 1$
$w_1 = w_2 = 0.5, m_1 = m_2 = 1000, h_1^2 = 10\%, h_2^2 = 10\%$	0.6	0.63
$w_1 = w_2 = 0.5, m_1 = m_2 = 1000, h_1^2 = 20\%, h_2^2 = 20\%$	0.64	0.67

S4 Table: Simulation results: effect of difference between baseline tissue-specific means of the phenotype (α_1, α_2) on the classification accuracy of eGST.
