# Supplementary material for: Leveraging eQTLs to identify individual-level tissue of interest for a complex trait
Source: PLoS Comput Biol. 2021 May 21;17(5):e1008915. doi: 10.1371/journal.pcbi.1008915 (PMC8174686; doi:10.1371/journal.pcbi.1008915)
Supplement: S5 Table — (PDF) [file pcbi.1008915.s013.pdf]

simulation scenario ($n = 40,000$)	AUC	
	$\mathrm{E}(\beta_{1j}) = \mathrm{E}(\beta_{2j}) = 0$	$\mathrm{E}(\beta_{1j}) = -0.2, \mathrm{E}(\beta_{2j}) = 0.2$
$w_1 = w_2 = 0.5, m_1 = m_2 = 1000, h_1^2 = 10\%, h_2^2 = 10\%$	0.6	0.63
$w_1 = w_2 = 0.5, m_1 = m_2 = 1000, h_1^2 = 20\%, h_2^2 = 20\%$	0.64	0.68

S5 Table: Simulation results: effect of difference between the mean of tissue-specific genetic effect size distribution on the classification accuracy of eGST. Here $j = 1, \ldots, m_1$ for β_1 (tissue 1) and $j = 1, \ldots, m_2$ for β_2 (tissue 2).
