# Supplementary material for: Leveraging eQTLs to identify individual-level tissue of interest for a complex trait
Source: PLoS Comput Biol. 2021 May 21;17(5):e1008915. doi: 10.1371/journal.pcbi.1008915 (PMC8174686; doi:10.1371/journal.pcbi.1008915)
Supplement: S6 Table — (PDF) [file pcbi.1008915.s014.pdf]

	First tissue-specific subtype posterior probability		
Tissue-specific subtype heritability	Group 1 with effect from tissue 1 mean (s.d.)	Group 2 with effect from both tissues mean (s.d.)	Group 3 with effect from tissue 2 mean (s.d.)
10%, 10%	52% (0.4%)	50% (0.4%)	48% (0.5%)
20%, 20%	55% (0.7%)	50% (0.7%)	45% (0.7%)
30%, 30%	57% (0.7%)	50% (0.7%)	42% (0.7%)
40%, 40%	60% (0.8%)	50% (0.9%)	39% (0.8%)
50%, 50%	63% (0.9%)	50% (1%)	36% (0.9%)

S6 Table: Pattern of first tissue-specific subtype posterior probability when a group of individuals have genetic effect from both tissues. Group 1 comprising 10K individuals have genetic effect only due to the first tissue-specific set of SNPs. Group 2 comprising the next set of 10K individuals have effect due to both of the tissue-specific set of SNPs. Group 3 comprising 10K individuals have effect only due to the second tissue-specific set of SNPs. Total sample size is 30,000.
