# Supplementary material for: Leveraging eQTLs to identify individual-level tissue of interest for a complex trait
Source: PLoS Comput Biol. 2021 May 21;17(5):e1008915. doi: 10.1371/journal.pcbi.1008915 (PMC8174686; doi:10.1371/journal.pcbi.1008915)
Supplement: S7 Table — (PDF) [file pcbi.1008915.s015.pdf]

65% threshold of posterior probability			
Heritability	Misclassified percentage mean (s.d.)	Correctly classified percentage, mean (s.d.)	Unclassified percentage mean (s.d.)
10%	3.4% (1.5%)	50.2% (14%)	46.4% (12.9%)
20%	1.4% (0.5%)	88.9% (2.8%)	9.7% (2.4%)
30%	0.9% (0.2%)	94.6% (1%)	4.5% (0.9%)
40%	1% (0.4%)	95.4% (2.1%)	3.6% (1.7%)
50%	1.4% (0.9%)	94.2% (4.1%)	4.3% (3.2%)
70% threshold of posterior probability			
Heritability	Misclassified percentage mean (s.d.)	Correctly classified percentage, mean (s.d.)	Unclassified percentage mean (s.d.)
10%	2.3% (1.1%)	29.3% (7.4%)	68.3% (6.5%)
20%	1% (0.3%)	81.5% (5.1%)	17.5% (4.9%)
30%	0.7% (0.1%)	91.9% (1.7%)	7.4% (1.6%)
40%	0.8% (0.3%)	93.5% (3.4%)	5.8% (3.1%)
50%	1.2% (0.7%)	92.1% (5.6%)	6.7% (4.9%)

S7 Table: Simulation results: Percentage of misclassification when one of the two tissues is completely irrelevant to the phenotype, i.e., the phenotype of all individuals have genetic effect only due to one tissue-specific set of SNPs. The misclassified percentage denotes the percentage of individuals classified to the irrelevant tissue. Here the first column provides the tissue-specific subtype heritability for the relevant tissue.
