# Supplementary material for: Leveraging eQTLs to identify individual-level tissue of interest for a complex trait
Source: PLoS Comput Biol. 2021 May 21;17(5):e1008915. doi: 10.1371/journal.pcbi.1008915 (PMC8174686; doi:10.1371/journal.pcbi.1008915)
Supplement: S8 Table — (PDF) [file pcbi.1008915.s016.pdf]

BMI bins	Number of individuals			
	adipose	brain	unassigned	total
12.81 - 14.67	1	6	2	9
14.67 - 16.52	30	69	49	148
16.52 - 18.37	272	441	577	1290
18.37 - 20.23	1146	1974	4689	7809
20.23 - 22.08	2021	3348	18551	23920
22.08 - 23.93	1157	1903	41738	44798
23.93 - 25.79	124	208	58906	59238
25.79 - 27.64	1	4	59284	59289
27.64 - 29.49	61	33	47808	47902
29.49 - 31.34	503	314	33062	33879
31.34 - 33.2	1154	719	19926	21799
33.2 - 35.05	1372	973	11368	13713
35.05 - 36.9	1186	877	6412	8475
36.9 - 38.76	899	748	3670	5317
38.76 - 40.61	639	551	2018	3208
40.61 - 42.46	454	396	1214	2064
42.46 - 44.32	307	270	694	1271
44.32 - 46.17	205	173	392	770
46.17 - 48.02	107	148	245	500
48.02 - 49.88	74	83	141	298
49.88 - 51.73	40	42	54	136
51.73 - 53.58	37	27	44	108
53.58 - 55.44	13	18	25	56
55.44 - 57.29	9	12	20	41
57.29 - 59.14	12	4	9	25
59.14 - 61	4	6	4	14
61 - 62.85	6	2	6	14
62.85 - 64.7	1	2	1	4
64.7 - 66.56	1	1	3	5
66.56 - 68.41	2	0	0	2

S8 Table: Number of individuals in consecutive non-overlapping bins of BMI who were assigned to adipose and brain specific subtype of BMI, and the number of individuals that remained unassigned by eGST based on 65% threshold of tissue-specific posterior probability.
