# Supplementary material for: Leveraging eQTLs to identify individual-level tissue of interest for a complex trait
Source: PLoS Comput Biol. 2021 May 21;17(5):e1008915. doi: 10.1371/journal.pcbi.1008915 (PMC8174686; doi:10.1371/journal.pcbi.1008915)
Supplement: S9 Table — (PDF) [file pcbi.1008915.s017.pdf]

WHRadjBMI bins	Number of individuals adipose	muscle	unassigned	total
-0.44 - -0.4	3	2	0	5
-0.4 - -0.36	4	2	2	8
-0.36 - -0.32	18	5	11	34
-0.32 - -0.28	50	14	28	92
-0.28 - -0.24	116	33	128	277
-0.24 - -0.21	369	107	473	949
-0.21 - -0.17	1134	301	2318	3753
-0.17 - -0.13	2201	660	10377	13238
-0.13 - -0.09	1311	410	29627	31348
-0.09 - -0.05	524	172	47369	48065
-0.05 - -0.01	1265	444	49017	50726
-0.01 - 0.03	1257	994	51773	54024
0.03 - 0.07	1077	1169	56769	59015
0.07 - 0.11	712	762	43180	44654
0.11 - 0.14	671	948	19153	20772
0.14 - 0.18	647	818	5285	6750
0.18 - 0.22	298	289	1162	1749
0.22 - 0.26	102	71	216	389
0.26 - 0.3	27	24	66	117
0.3 - 0.34	11	9	11	31
0.34 - 0.38	2	3	4	9
0.38 - 0.42	1	0	1	2
0.42 - 0.46	1	0	0	1
0.46 - 0.49	0	1	2	3
0.49 - 0.53	0	0	1	1
0.53 - 0.57	0	0	0	0
0.57 - 0.61	0	0	1	1
0.61 - 0.65	0	0	0	0
0.65 - 0.69	0	0	0	0
0.69 - 0.73	1	0	0	1

S9 Table: Number of individuals in consecutive non-overlapping bins of WHRadjBMI who were assigned to adipose and muscle specific subtype of WHRadjBMI, and the number of individuals that remained unassigned by eGST based on 65% threshold of tissue-specific posterior probability.
