# Supplementary material for: Leveraging eQTLs to identify individual-level tissue of interest for a complex trait
Source: PLoS Comput Biol. 2021 May 21;17(5):e1008915. doi: 10.1371/journal.pcbi.1008915 (PMC8174686; doi:10.1371/journal.pcbi.1008915)
Supplement: S10 Table — (PDF) [file pcbi.1008915.s018.pdf]

| Category | Trait | adipose P | brain P | Means adipose | brain | popln | signif tissue |
|---|---|---|---|---|---|---|---|
| Body size measures | Body mass index | 1.84E-227 | 3.01E-230 | 30.39 | 27.60 | 27.40 | both |
| Body size measures | Waist circumference | 1.06E-183 | 1.46E-108 | 95.50 | 89.20 | 90.33 | both |
| Body size measures | Weight | 8.66E-175 | 2.82E-140 | 85.20 | 77.58 | 78.31 | both |
| Body size measures | Standing height | 4.32E-64 | 2.49E-56 | 167.45 | 167.65 | 168.84 | both |
| Body size measures | Sitting height | 9.28E-28 | 2.14E-53 | 88.97 | 88.82 | 89.41 | both |
| Blood count | Haemoglobin concentration | 1.79E-48 | 3.94E-122 | 14.04 | 13.97 | 14.21 | both |
| Blood count | Haematocrit percentage | 3.60E-33 | 4.67E-98 | 40.76 | 40.54 | 41.16 | both |
| Blood count | Red blood cell erythrocyte count | 6.27E-14 | 2.90E-96 | 4.48 | 4.44 | 4.51 | both |
| Blood count | High light scatter reticulocyte percentage | 3.58E-67 | 1.54E-21 | 0.44 | 0.40 | 0.40 | both |
| Blood count | Red blood cell erythrocyte distribution width | 9.82E-62 | 1.26E-24 | 13.62 | 13.56 | 13.47 | both |
| Blood count | White blood cell leukocyte count | 1.84E-58 | 6.44E-06 | 7.15 | 6.98 | 6.89 | both |
| Blood count | High light scatter reticulocyte count | 1.09E-56 | 4.95E-37 | 0.02 | 0.02 | 0.02 | both |
| Blood count | Neutrophill count | 1.34E-55 | 1.47E-10 | 4.45 | 4.35 | 4.24 | both |
| Blood count | Reticulocyte count | 1.92E-32 | 3.69E-54 | 0.06 | 0.06 | 0.06 | both |
| Blood count | Reticulocyte percentage | 8.06E-44 | 2.39E-30 | 1.42 | 1.32 | 1.35 | both |
| Blood count | Mean corpuscular haemoglobin concentration | 2.91E-22 | 1.78E-15 | 34.46 | 34.48 | 34.54 | both |
| Blood count | Platelet crit | 1.92E-16 | 5.08E-07 | 0.24 | 0.23 | 0.23 | both |
| Blood count | Basophill count | 1.31E-13 | 7.98E-06 | 0.04 | 0.04 | 0.03 | both |
| Blood count | Monocyte percentage | 1.77E-13 | 2.29E-13 | 6.99 | 6.97 | 7.10 | both |
| Blood count | Neutrophill percentage | 1.18E-12 | 2.01E-12 | 61.67 | 61.67 | 61.14 | both |
| Blood count | Mean corpuscular volume | 2.42E-11 | 2.74E-05 | 91.09 | 91.49 | 91.34 | both |
| Blood count | Platelet count | 8.36E-10 | 1.94E-05 | 256.95 | 255.58 | 253.21 | both |
| Blood count | Lymphocyte percentage | 1.46E-09 | 1.56E-08 | 28.20 | 28.25 | 28.63 | both |
| Blood count | Monocyte count | 7.85E-07 | 0.0002 | 0.49 | 0.47 | 0.48 | both |
| Blood count | Basophill percentage | 1.38E-05 | 1.86E-05 | 0.59 | 0.60 | 0.57 | both |
| Medications | Number of treatments medications taken | 6.17E-102 | 7.84E-11 | 3.08 | 2.70 | 2.45 | both |
| Medical conditions | Number of self reported non cancer illnesses | 1.32E-84 | 6.03E-06 | 2.24 | 1.98 | 1.87 | both |
| Medical conditions | Interpolated Age of participant when non cancer illness first diagnosed | 3.77E-05 | 6.52E-09 | 41.89 | 41.56 | 42.32 | both |
| Baseline characteristics | Townsend deprivation index at recruitment | 1.26E-53 | 6.69E-28 | -1.13 | -1.26 | -1.58 | both |
| Mental health | Neuroticism score | 1.41E-24 | 5.79E-19 | 4.44 | 4.38 | 4.10 | both |
| Urine assays | Creatinine enzymatic in urine | 1.51E-05 | 2.80E-24 | 9077.69 | 8429.45 | 8806.93 | both |
| Urine assays | Sodium in urine | 6.09E-13 | 3.67E-08 | 79.56 | 74.72 | 76.21 | both |
| Sleep | Sleep duration | 0.0001 | 1.92E-06 | 7.08 | 7.08 | 7.13 | both |
| Blood count | Immature reticulocyte fraction | 2.46E-69 | 0.13 | 0.30 | 0.29 | 0.29 | adipose |
| Blood count | Mean corpuscular haemoglobin | 5.73E-25 | 0.96 | 31.39 | 31.55 | 31.55 | adipose |
| Blood count | Lymphocyte count | 2.47E-15 | 0.26 | 1.98 | 1.93 | 1.95 | adipose |
| Blood count | Eosinophill count | 8.62E-07 | 0.02 | 0.18 | 0.17 | 0.17 | adipose |
| Medical conditions | Non cancer illness code self reported | 2.02E-35 | 0.05 | 2405.55 | 2779.19 | 2891.97 | adipose |
| Medical conditions | Non cancer illness year age first occurred | 1.25E-15 | 0.002 | 628.73 | 703.82 | 702.47 | adipose |
| Blood count | Mean sphered cell volume | 0.05 | 9.57E-21 | 82.86 | 83.37 | 82.87 | brain |
| Blood count | Mean reticulocyte volume | 0.09 | 2.40E-08 | 106.00 | 106.22 | 105.83 | brain |
| Blood count | Eosinophill percentage | 0.37 | 3.26E-07 | 2.53 | 2.52 | 2.56 | brain |
| Blood count | Platelet distribution width | 0.79 | 3.69E-06 | 16.49 | 16.47 | 16.49 | brain |
| Urine assays | Potassium in urine | 0.18 | 8.26E-12 | 63.61 | 61.68 | 63.47 | brain |
| Early life factors | Birth weight | 0.001 | 4.19E-06 | 3.30 | 3.30 | 3.33 | brain |

**S10 Table**: Quantitative traits among 106 phenotypes in UK Biobank which are differentially distributed between the adipose (and/or brain) specific subtype group of individuals for BMI and the remaining population. We provide the p-values of testing heterogeneity of each trait between each tissue-specific subtype group of individuals and the remaining population. For each trait, the tissue-specific (adipose and brain) mean which is calculated only in the individuals classified as the corresponding tissue-specific subtype of BMI are provided. We also provide the trait means computed in whole sample (popln). For each trait, we list the tissues for which the trait was differentially distributed between the corresponding tissue-specific subtype group of individuals and the remaining population (signif tissue).
