# Supplementary material for: Leveraging eQTLs to identify individual-level tissue of interest for a complex trait
Source: PLoS Comput Biol. 2021 May 21;17(5):e1008915. doi: 10.1371/journal.pcbi.1008915 (PMC8174686; doi:10.1371/journal.pcbi.1008915)
Supplement: S11 Table — (PDF) [file pcbi.1008915.s019.pdf]

Category	Trait	AS P	MS P	Means			signif tissue
				AS	MS	popln	
Body size measure	WHRadjBMI	0	2.37E-192	-0.04	0.03	0.00	both
Body size measure	WHR	3.68E-232	3.71E-201	0.85	0.91	0.87	both
Body size measures	Body mass index	6.76E-147	4.07E-42	29.19	28.41	27.39	both
Body size measures	Standing height	2.86E-124	1.38E-37	166.91	167.48	168.84	both
Body size measures	Sitting height	1.93E-70	1.18E-55	88.68	88.53	89.41	both
Body size measures	Weight	1.13E-36	9.02E-07	81.27	79.83	78.31	both
Blood count	Haemoglobin concentration	2.27E-81	6.20E-11	14.01	14.12	14.21	both
Blood count	Haematocrit percentage	1.08E-59	1.03E-06	40.67	40.96	41.16	both
Blood count	Red blood cell erythrocyte distribution width	6.69E-40	3.06E-14	13.57	13.56	13.47	both
Blood count	Red blood cell erythrocyte count	8.69E-38	4.27E-06	4.47	4.49	4.51	both
Blood count	High light scatter reticulocyte percentage	5.46E-06	7.63E-31	0.41	0.43	0.40	both
Blood count	White blood cell leukocyte count	3.41E-06	1.64E-28	6.99	7.12	6.89	both
Blood count	Immature reticulocyte fraction	3.27E-17	1.74E-23	0.29	0.30	0.29	both
Blood count	Neutrophill count	1.01E-06	3.91E-22	4.32	4.41	4.24	both
Blood count	Mean corpuscular haemoglobin concentration	1.44E-21	2.67E-08	34.47	34.47	34.54	both
Blood count	Monocyte percentage	2.51E-21	6.52E-10	6.94	6.98	7.10	both
Blood count	Platelet crit	5.69E-18	1.54E-11	0.24	0.24	0.23	both
Blood count	Lymphocyte count	0.0003	1.30E-15	1.98	2.00	1.95	both
Blood count	Platelet count	8.60E-08	4.87E-08	256.00	257.00	253.21	both
Medications	Number of treatments medications taken	9.24E-30	4.17E-50	2.77	2.97	2.45	both
Medical conditions	Number of self reported non cancer illnesses	5.95E-26	1.19E-34	2.08	2.15	1.86	both
Baseline characteristics	Townsend deprivation index at recruitment	3.34E-10	8.09E-17	-1.41	-1.26	-1.58	both
Mental health	Neuroticism score	1.72E-06	2.04E-08	4.24	4.35	4.10	both
Blood count	Mean corpuscular haemoglobin	1.64E-18	0.03	31.43	31.50	31.55	adipose
Blood count	Monocyte count	4.67E-08	0.04	0.47	0.49	0.48	adipose
Blood count	Mean corpuscular volume	9.40E-06	0.74	91.18	91.38	91.34	adipose
Blood count	Mean platelet thrombocyte volume	9.74E-05	0.48	9.36	9.33	9.32	adipose
Blood count	Eosinophill percentage	0.0004	0.54	2.49	2.53	2.56	adipose
Urine assays	Creatinine enzymatic in urine	0.0001	0.02	8633.55	8618.76	8806.64	adipose
Body size measures	Waist circumference	0.20	3.17E-127	90.80	94.50	90.33	muscle
Blood count	High light scatter reticulocyte count	0.01	2.98E-25	0.02	0.02	0.02	muscle
Blood count	Reticulocyte percentage	0.14	2.25E-23	1.36	1.42	1.35	muscle
Blood count	Reticulocyte count	0.22	2.65E-17	0.06	0.06	0.06	muscle
Blood count	Basophill count	0.27	4.63E-10	0.03	0.04	0.03	muscle
Medical conditions	Non cancer illness code self reported	0.0007	1.21E-10	3071.24	2623.36	2891.68	muscle
Medical conditions	Non cancer illness year age first occurred	0.06	4.69E-06	689.59	631.65	702.50	muscle
Education	Age completed full time education	0.12	8.65E-06	16.36	16.25	16.45	muscle

S11 Table: Quantitative traits among 106 phenotypes in UK Biobank which are differentially distributed between the adipose subcutaneous (AS) (and/or muscle skeletal (MS)) specific subtype group of individuals for WHRadjBMI and the remaining population. We provide the p-values of testing heterogeneity of each trait between each tissue-specific subtype group of individuals and the remaining population. For each trait, the tissue-specific (AS and MS) mean which is calculated only in the individuals classified as the corresponding tissue-specific subtype of WHRadjBMI are provided. We also provide the trait means computed in whole sample (popln). For each trait, we list the tissues for which the trait was differentially distributed between the corresponding tissue-specific subtype group of individuals and the remaining population (signif tissue).
