# Supplementary material for: Leveraging eQTLs to identify individual-level tissue of interest for a complex trait
Source: PLoS Comput Biol. 2021 May 21;17(5):e1008915. doi: 10.1371/journal.pcbi.1008915 (PMC8174686; doi:10.1371/journal.pcbi.1008915)
Supplement: S12 Table — (PDF) [file pcbi.1008915.s020.pdf]

Trait	P adipose		P brain		Risk			signif tissue	
	primary	BMIadj	primary	BMIadj	adipose	brain	popln	primary	BMIadj
*Sex(male/female)	1.20E-83	3.06E-143	8.31E-129	3.95E-135	0.38	0.36	0.46	both	both
*Diabetes diagnosed by doctor	3.38E-90	0.0004	2.73E-30	0.003	0.09	0.07	0.05	both	adipose
*Long standing illness disability or infirmity	4.77E-82	1.89E-07	1.80E-27	2.54E-16	0.41	0.37	0.32	both	both
*Fed up feelings	3.04E-41	9.07E-13	2.61E-08	1.32E-06	0.47	0.43	0.41	both	both
*Loneliness isolation	1.37E-38	8.82E-16	5.88E-20	6.80E-16	0.22	0.21	0.18	both	both
*Other serious medical condition disability diagnosed by doctor	2.32E-26	3.17E-05	3.54E-13	3.33E-09	0.24	0.23	0.20	both	both
*Miserableness	5.70E-30	8.05E-17	5.03E-09	2.28E-08	0.48	0.45	0.43	both	both
*Tense highly strung	1.14E-05	9.67E-12	2.09E-23	9.45E-23	0.19	0.20	0.17	both	both
*Mood swings	2.17E-24	3.13E-09	6.58E-06	3.06E-05	0.50	0.47	0.45	both	both
*Seen doctor GP for nerves anxiety tension or depression	2.20E-23	4.56E-12	2.31E-12	2.42E-11	0.39	0.37	0.34	both	both
*Snoring	8.37E-06	1.03E-28	6.78E-16	8.28E-29	0.40	0.34	0.37	both	both
*Seen a psychiatrist for nerves anxiety tension or depression	4.27E-18	3.24E-09	1.21E-10	3.83E-09	0.14	0.13	0.11	both	both
*Worrier anxious feelings	2.80E-07	1.55E-20	4.45E-16	1.18E-17	0.59	0.60	0.57	both	both
*Sensitivity hurt feelings	2.70E-13	5.29E-15	4.55E-10	3.47E-10	0.59	0.58	0.56	both	both
*Guilty feelings	2.90E-13	2.27E-15	1.93E-08	1.50E-08	0.31	0.30	0.28	both	both
*Suffer from nerves	4.25E-05	1.15E-07	1.90E-05	1.57E-05	0.23	0.23	0.21	both	both
*Nervous feelings	0.001	4.55E-16	2.01E-22	1.93E-19	0.25	0.27	0.24	brain	both
*Risk taking	0.018	3.59E-08	9.94E-11	4.25E-12	0.24	0.23	0.25	brain	both
*Worry too long after embarrassment	0.001	4.08E-11	8.40E-06	2.72E-06	0.49	0.50	0.48	brain	both

S12 Table: Case-control traits among 106 phenotypes considered in the UK biobank which are differentially distributed between the adipose (and/or brain) specific subtype group of individuals for BMI and the remaining population. For each trait, we provide the p-value of testing heterogeneity between each tissue-specific subtype group of individuals and the remaining population before (primary) and after BMI adjustment (BMIadj). For each trait, tissue-specific groups which appear to be significantly heterogeneous (signif tissue) before (primary) and after BMI adjustment (BMIadj) are provided. The asterisk mark attached to the traits indicate which trait remains differentially distributed between at least one of the tissue-specific groups and the remaining population after BMI adjustment. Tissue-specific risk of the disorders along with population-level risk are also provided.
