# Supplementary material for: Leveraging eQTLs to identify individual-level tissue of interest for a complex trait
Source: PLoS Comput Biol. 2021 May 21;17(5):e1008915. doi: 10.1371/journal.pcbi.1008915 (PMC8174686; doi:10.1371/journal.pcbi.1008915)
Supplement: S13 Table — (PDF) [file pcbi.1008915.s021.pdf]

Trait	P AS		P MS		Risk			signif tissue	
	primary	adjusted	primary	adjusted	AS	MS	popln	primary	adjusted
*Sex(male/female)	4.37E-170	1.14E-08	6.69E-52	0	0.34	0.37	0.46	both	both
*Diabetes diagnosed by doctor	5.42E-13	2.68E-22	8.91E-40	4.26E-11	0.06	0.08	0.05	both	both
*Long standing illness disability or infirmity	4.69E-21	3.51E-50	2.92E-25	6.61E-12	0.36	0.38	0.32	both	both
*Seen doctor GP for nerves anxiety tension or depression	8.80E-16	0.01	2.73E-12	5.68E-22	0.38	0.38	0.34	both	muscle
*Loneliness isolation	1.02E-07	0.002	1.81E-14	2.61E-18	0.20	0.21	0.18	both	muscle
*Miserableness	2.54E-11	0.09	2.37E-08	3.04E-16	0.46	0.46	0.43	both	muscle
*Fed up feelings	2.59E-10	1.90E-07	8.04E-08	2.24E-09	0.43	0.44	0.41	both	both
*Other serious medical condition disability diagnosed by doctor	3.83E-09	2.61E-16	2.36E-07	0.0002	0.23	0.23	0.20	both	both
*Seen a psychiatrist for nerves anxiety tension or depression	1.70E-05	6.28E-06	6.95E-10	1.21E-09	0.13	0.14	0.11	both	both
Ever smoked	4.74E-06	0.004	0.0004	0.88	0.58	0.62	0.60	both	none
*Guilty feelings	0.0002	0.05	1.57E-06	5.14E-13	0.30	0.31	0.28	both	muscle
*Sensitivity hurt feelings	2.69E-05	0.02	0.0002	2.37E-14	0.58	0.58	0.56	both	muscle
*Mood swings	2.01E-05	0.002	2.90E-05	1.65E-06	0.47	0.48	0.45	both	muscle
*Risk taking	8.17E-10	0.30	0.34	1.90E-07	0.23	0.25	0.25	adipose	muscle
Worry too long after embarrassment	3.26E-06	0.48	0.89	0.003	0.50	0.48	0.48	adipose	none
*Worrier anxious feelings	1.94E-05	0.38	0.002	7.28E-10	0.59	0.59	0.57	adipose	muscle
Pregnant	0.02	0.13	2.43E-06	0.01	0.001	0.002	0.0004	muscle	none

S13 Table: Case-control traits among 106 phenotypes considered in the UK biobank which are differentially distributed between the adipose subcutaneous (AS) (and/or muscle skeletal (MS)) specific subtype group of individuals for WHRadjBMI and the remaining population. For each trait, we provide the p-value of testing heterogeneity between each tissue-specific subtype group of individuals and the remaining population before (primary) and after WHRadjBMI adjustment (adjusted). For each trait, tissue-specific groups which appear to be significantly heterogeneous (signif tissue) before (primary) and after WHRadjBMI adjustment (adjusted) are provided. The asterisk mark attached to the traits indicate which trait remains differentially distributed between at least one of the tissue-specific groups and the remaining population after WHRadjBMI adjustment. Tissue-specific risk of the disorders along with population-level risk are also provided.
