# Supplementary material for: Leveraging eQTLs to identify individual-level tissue of interest for a complex trait
Source: PLoS Comput Biol. 2021 May 21;17(5):e1008915. doi: 10.1371/journal.pcbi.1008915 (PMC8174686; doi:10.1371/journal.pcbi.1008915)
Supplement: S14 Table — (PDF) [file pcbi.1008915.s022.pdf]

Trait	P AS		P MS		signif tissue		#categ
	primary	adjusted	primary	adjusted	primary	adjusted	
*Alcohol intake frequency	8.90E-74	1.41E-07	3.66E-24	6.98E-11	both	both	6
*Overall health rating	2.35E-46	2.11E-23	6.34E-49	3.35E-11	both	both	4
*Weight change compared with 1 year ago	8.51E-37	6.67E-19	2.89E-17	5.46E-27	both	both	3
*Alcohol drinker status	2.93E-23	4.56E-14	7.33E-06	9.74E-09	both	both	3
*Falls in the last year	1.43E-23	4.22E-12	4.68E-14	1.95E-10	both	both	3
Frequency of tiredness lethargy in last weeks	3.69E-22	0.002	3.00E-17	0.12	both	none	4
*Current tobacco smoking	0.0004	0.35	9.55E-19	4.74E-05	both	muscle	3
Qualifications	0.0002	0.1	5.74E-17	1	both	none	7
*Smoking status	1.46E-05	0.35	1.29E-16	4.50E-05	both	muscle	3
*Sleeplessness insomnia	7.08E-13	0.02	1.02E-09	3.51E-10	both	muscle	3
*Frequency of depressed mood in last 2 weeks	2.94E-08	0.0002	2.60E-05	0.009	both	adipose	4
*Blood clot DVT bronchitis emphysema asthma rhinitis eczema allergy diagnosed by doctor	3.07E-06	0.07	1.12E-10	1.57E-06	both	muscle	6
*Getting up in morning	2.40E-06	0.0002	2.97E-11	0.54	both	adipose	4
*Frequency of unenthusiasm disinterest in last 2 weeks	2.23E-05	2.54E-05	5.29E-07	0.0009	both	adipose	4
Daytime dozing sleeping narcolepsy	3.88E-06	0.16	0.0003	0.46	both	none	4
Illness injury bereavement stress in last 2 years	1.55E-09	0.06	0.003	0.42	adipose	none	7
Past tobacco smoking	8.52E-06	0.02	0.004	0.24	adipose	none	4
*Nap during day	0.12	1.77E-18	2.65E-10	0.14	muscle	adipose	3
Frequency of tenseness restlessness in last 2 weeks	0.02	0.01	3.04E-06	0.05	muscle	none	4
Morning evening person chronotype	0.001	0.03	0.0001	0.43	muscle	none	4

S14 Table: Qualitative/categorical traits with three or more categories that are differentially distributed between at least one of adipose subcutaneous (AS) and muscle skeletal (MS) specific subtype groups of individuals for WHRadjBMI and the remaining population. For each trait we provide the p-values of testing heterogeneity between each tissue-specific subtype group of individuals and the remaining population before (primary) and after WHRadjBMI adjustment (adjusted). For each trait, tissue-specific groups which appear to be significantly heterogeneous (signif tissue) before (primary) and after WHRadjBMI adjustment (adjusted) are also provided. The asterisk mark attached to the traits indicate which trait remains differentially distributed between at least one of the tissue-specific groups and the remaining population after WHRadjBMI adjustment. The number of categories for each trait (#categ) are also listed.
