# Supplementary material for: Leveraging eQTLs to identify individual-level tissue of interest for a complex trait
Source: PLoS Comput Biol. 2021 May 21;17(5):e1008915. doi: 10.1371/journal.pcbi.1008915 (PMC8174686; doi:10.1371/journal.pcbi.1008915)
Supplement: S15 Table — (PDF) [file pcbi.1008915.s023.pdf]

Trait	adipose P	brain P	signif tissue	Tissue-specific relative change adipose BMIadj	adipose primary	brain BMIadj	brain primary
Non cancer illness code self reported	0	3.37E-06	both	-1.46	-3.95	-0.26	-0.92
Interpolated Age of participant when non cancer illness first diagnosed	3.05E-22	4.31E-14	both	-7.14	-2.43	-5.53	-4.28
Number of self reported non cancer illnesses	3.01E-13	1.60E-21	both	7.22	21.14	5.87	6.71
Haemoglobin concentration	8.50E-118	4.50E-125	both	-22.72	-14.19	-20.99	-20.23
Red blood cell erythrocyte count	2.98E-93	8.65E-108	both	-19.83	-7.54	-19.81	-18.61
Haematocrit percentage	1.52E-94	1.57E-102	both	-20.01	-11.54	-18.92	-18.18
Reticulocyte count	1.70E-31	1.24E-42	both	-7.00	8.54	-6.46	-5.06
Mean sphered cell volume	3.28E-16	2.44E-27	both	8.84	-0.23	10.69	9.89
Red blood cell erythrocyte distribution width	1.17E-22	3.01E-25	both	11.49	16.74	9.86	10.18
Mean corpuscular haemoglobin concentration	1.39E-23	5.61E-15	both	-8.11	-7.86	-5.97	-5.94
Reticulocyte percentage	5.39E-15	1.46E-23	both	-4.37	8.85	-4.00	-2.90
Monocyte percentage	1.31E-15	2.68E-13	both	-4.55	-4.09	-5.25	-5.23
Neutrophill percentage	4.68E-13	3.96E-12	both	6.72	6.58	6.62	6.61
High light scatter reticulocyte count	1.16E-10	3.16E-08	both	-5.96	19.22	-5.62	-3.31
Neutrophill count	2.40E-08	2.43E-10	both	6.02	15.43	7.03	7.61
Basophill percentage	1.31E-09	6.19E-05	both	5.06	4.48	4.85	4.77
Mean reticulocyte volume	6.71E-06	4.40E-09	both	4.74	2.28	5.38	5.18
Lymphocyte percentage	3.06E-08	2.12E-08	both	-5.54	-6.01	-5.40	-5.43
Platelet crit	2.29E-08	7.27E-07	both	5.83	8.44	4.31	4.50
Eosinophill percentage	5.59E-05	5.25E-08	both	-3.43	-1.46	-2.49	-2.35
Platelet count	1.22E-05	2.66E-05	both	4.78	6.48	4.01	4.13
Waist circumference	6.43E-106	1.15E-118	both	-22.89	39.90	-21.34	-8.71
Weight	7.13E-71	1.60E-66	both	-17.15	45.05	-15.66	-4.81
Standing height	6.79E-58	6.24E-63	both	-14.74	-15.57	-13.37	-13.43
Sitting height	2.15E-46	8.76E-48	both	-13.52	-9.42	-13.10	-12.79
Number of treatments medications taken	6.14E-17	9.61E-38	both	9.72	24.52	8.89	9.68
Neuroticism score	6.29E-14	4.23E-31	both	10.52	10.86	9.01	9.02
Townsend deprivation index at recruitment	7.86E-20	7.03E-29	both	9.85	15.63	11.01	11.34
Creatinine enzymatic in urine	1.36E-20	1.07E-13	both	-8.62	4.89	-7.73	-6.85
Potassium in urine	3.55E-10	1.11E-11	both	-6.64	0.42	-5.90	-5.49
Birth weight	3.63E-07	5.43E-05	both	-5.99	-3.70	-4.81	-4.65
Non cancer illness year age first occurred	1.67E-90	0.25	adipose	2.31	-8.27	2.97	0.15
Monocyte count	1.82E-07	0.003	adipose	-2.23	5.21	-2.95	-2.42
Eosinophill count	3.15E-05	0.18	adipose	-2.45	3.56	-0.87	-0.44
High light scatter reticulocyte percentage	6.41E-05	0.003	adipose	-2.51	12.78	-2.19	-1.00
Sodium in urine	1.45E-08	0.0005	adipose	-5.73	7.97	-4.45	-3.55
Basophill count	0.004	1.61E-22	brain	5.34	7.50	5.18	5.30
Platelet distribution width	0.002	2.59E-06	brain	-2.92	0.44	-4.49	-4.24
Mean corpuscular volume	0.07	4.19E-06	brain	1.93	-5.86	4.09	3.49
White blood cell leukocyte count	0.001	1.56E-05	brain	2.67	13.43	3.95	4.67
Sleep duration	0.01	1.55E-54	brain	-2.33	-4.26	-4.08	-4.20

S15 Table: Heterogeneity of non-BMI quantitative traits between the adipose (brain) specific subtype group of individuals for BMI and the remaining population after BMI adjustment of the traits in the population using linear regression. For each trait, we provide the p-values of testing heterogeneity between each tissue-specific subtype group of individuals and the remaining population after BMI adjustment. For each trait, tissue-specific groups which appear to be significantly heterogeneous (signif tissue) after BMI adjustment are provided. To measure the primary tissue-specific relative change of a trait we calculate the following: $\frac{\text{tissue specific mean - remaining population mean}}{\text{population s.d.}} \times 100$, where the tissue-specific mean is computed only in the individuals with the corresponding tissue-specific subtype of BMI. The same measure is calculated for a trait residual obtained after adjusting for BMI in the population to quantify the tissue-specific relative change of the trait after BMI adjustment (BMIadj).
