# Supplementary material for: Leveraging eQTLs to identify individual-level tissue of interest for a complex trait
Source: PLoS Comput Biol. 2021 May 21;17(5):e1008915. doi: 10.1371/journal.pcbi.1008915 (PMC8174686; doi:10.1371/journal.pcbi.1008915)
Supplement: S16 Table — (PDF) [file pcbi.1008915.s024.pdf]

Trait	AS P	MS P	signif tissue	Tissue-specific relative change			
				AS		MS	
				adjusted	primary	adjusted	primary
Non cancer illness code self reported	1.66E-231	7.82E-110	both	0.41	1.46	-1.36	-2.15
Non cancer illness year age first occurred	5.06E-93	1.00E-26	both	-5.69	-1.45	-4.67	-7.83
Number of self reported non cancer illnesses	1.94E-87	6.28E-13	both	15.63	11.79	13.14	15.63
Standing height	1.81E-08	5.60E-178	both	3.89	-21.63	-36.75	-14.98
Sitting height	8.24E-06	1.99E-160	both	3.22	-15.58	-33.73	-18.68
Body mass index	6.65E-147	4.13E-42	both	40.01	39.22	21.98	21.86
Weight	1.09E-132	9.16E-08	both	35.38	19.36	0.19	9.81
Waist circumference	6.93E-113	2.32E-18	both	35.87	3.68	15.89	31.76
Haemoglobin concentration	1.06E-22	1.61E-98	both	7.97	-16.95	-27.44	-7.94
Haematocrit percentage	3.37E-27	7.59E-75	both	9.23	-14.40	-23.49	-5.65
Red blood cell erythrocyte count	1.14E-23	6.46E-59	both	8.78	-11.66	-20.76	-5.71
High light scatter reticulocyte count	2.01E-38	5.32E-07	both	12.12	3.64	6.41	11.99
Red blood cell erythrocyte distribution width	1.28E-31	5.61E-17	both	10.05	11.09	10.83	10.08
Immature reticulocyte fraction	3.84E-31	1.12E-16	both	11.45	8.25	10.55	12.68
High light scatter reticulocyte percentage	5.11E-30	5.07E-16	both	6.84	3.42	8.27	10.43
White blood cell leukocyte count	7.27E-24	3.21E-17	both	9.32	5.10	8.81	11.61
Neutrophill count	7.85E-24	1.22E-12	both	10.13	5.68	9.32	12.25
Basophill count	3.48E-11	1.37E-23	both	1.84	2.28	5.89	5.58
Monocyte count	4.62E-18	1.92E-06	both	6.49	-2.36	-2.72	3.28
Mean corpuscular haemoglobin concentration	3.22E-05	2.08E-17	both	-2.73	-7.16	-9.84	-6.78
Reticulocyte percentage	4.03E-17	2.22E-10	both	4.94	1.30	6.24	8.64
Number of treatments medications taken	3.89E-81	2.33E-27	both	15.67	12.53	17.78	19.77
Townsend deprivation index at recruitment	1.43E-19	2.17E-12	both	7.92	5.76	9.42	10.87
Creatinine enzymatic in urine	1.27E-27	1.42E-27	both	8.87	-3.13	-11.60	-3.35
Reticulocyte count	1.24E-24	0.02	adipose	6.39	-0.24	2.40	6.86
Age completed full time education	3.06E-20	0.04	adipose	-4.91	-3.10	-5.79	-7.13
Platelet crit	0.01	3.06E-35	muscle	-1.96	7.53	14.62	7.93
Monocyte percentage	0.29	5.34E-32	muscle	1.21	-6.11	-9.67	-4.58
Platelet count	0.002	1.37E-21	muscle	-2.26	4.84	11.42	6.49
Lymphocyte count	0.006	6.57E-17	muscle	2.35	2.59	4.72	4.58
Mean corpuscular haemoglobin	0.02	1.24E-07	muscle	-2.05	-7.16	-6.43	-2.91
Neuroticism score	0.90	5.28E-15	muscle	0.24	4.48	10.63	7.91

S16 Table: Phenotypic heterogeneity of non-WHR quantitative traits between adipose subcutaneous (AS) (muscle skeletal (MS)) specific group of individuals for WHRadjBMI and the remaining population after WHRadjBMI adjustment of the traits in the population using linear regression. For each trait we provide the p-values of testing heterogeneity for the two tissues after WHRadjBMI adjustment. For each trait, tissues which appear to be significant (signif tissue) after WHRadjBMI adjustment are provided. To measure the primary tissue-specific relative change of a trait we calculate the following: $\frac{\text{tissue-specific mean - remaining population mean}}{\text{population s.d.}} \times 100$, where the tissue-specific mean is computed based only on the individuals classified as the corresponding tissue-specific subtype of WHRadjBMI. The same measure is calculated for the trait residual obtained after adjusting for WHRadjBMI in the population to quantify the tissue-specific relative change of the trait after WHRadjBMI adjustment (adjusted).
