# Supplementary material for: Leveraging eQTLs to identify individual-level tissue of interest for a complex trait
Source: PLoS Comput Biol. 2021 May 21;17(5):e1008915. doi: 10.1371/journal.pcbi.1008915 (PMC8174686; doi:10.1371/journal.pcbi.1008915)
Supplement: S17 Table — (PDF) [file pcbi.1008915.s025.pdf]

Trait	BMI matched adip change		adip change	BMI matched brain change		brain change
	mean	sd	primary	mean	sd	primary
Haemoglobin concentration	0.78	0.58	14.19	3.50	0.77	20.23
Mean sphered cell volume	1.68	0.87	0.23	2.44	0.79	9.89
Mean reticulocyte volume	1.93	0.89	2.28	2.04	0.79	5.18
Number of self reported non cancer illnesses	2.91	0.92	21.14	0.90	0.64	6.71
Mean corpuscular volume	1.05	0.72	5.86	2.69	0.83	3.49
Interpolated Age of participant when non cancer illness first diagnosed	0.81	0.57	2.43	2.89	0.92	4.28
Haematocrit percentage	0.71	0.53	11.54	2.83	0.78	18.18
Standing height	0.72	0.52	15.57	2.74	0.74	13.43
Number of treatments medications taken	2.60	0.92	24.52	0.81	0.60	9.68
Mean corpuscular haemoglobin concentration	0.87	0.68	7.86	2.49	0.82	5.94
Red blood cell erythrocyte distribution width	2.31	0.92	16.74	0.91	0.66	10.18
Neuroticism score	1.73	0.89	10.86	1.24	0.74	9.02
Non cancer illness year age first occurred	1.44	0.79	8.27	1.39	0.80	0.15
Non cancer illness code self reported	1.69	0.85	3.95	0.91	0.64	0.92
Red blood cell erythrocyte count	0.86	0.61	7.54	1.43	0.74	18.61
Birth weight	1.43	0.95	3.70	0.84	0.62	4.65
Lymphocyte percentage	1.38	0.82	6.01	0.82	0.58	5.43
Potassium in urine	0.71	0.53	0.42	1.45	0.78	5.49
Sleep duration	0.75	0.58	4.26	1.39	0.75	4.20
Eosinophill count	0.89	0.68	3.56	1.22	0.76	0.44
Platelet crit	1.10	0.75	8.44	1.00	0.68	4.50
Neutrophill count	0.98	0.72	15.43	1.10	0.73	7.61
Sitting height	0.86	0.63	9.42	1.10	0.67	12.79
Waist circumference	0.49	0.35	39.90	1.46	0.49	8.71
Eosinophill percentage	0.72	0.55	1.46	1.19	0.74	2.35
High light scatter reticulocyte percentage	1.10	1.06	12.78	0.76	0.72	1.00
Sodium in urine	0.79	0.58	7.97	1.06	0.66	3.55
Reticulocyte count	0.73	0.55	8.54	1.09	0.63	5.06
Monocyte count	1.03	0.71	5.21	0.77	0.57	2.42
Neutrophill percentage	1.07	0.75	6.58	0.70	0.51	6.61
Platelet count	1.04	0.71	6.48	0.70	0.52	4.13
Creatinine enzymatic in urine	0.91	0.68	4.89	0.84	0.60	6.85
Monocyte percentage	0.97	0.70	4.09	0.76	0.55	5.23
Platelet distribution width	0.95	0.66	0.44	0.75	0.54	4.24
Reticulocyte percentage	0.80	0.61	8.85	0.85	0.57	2.90
Basophill count	0.83	0.64	7.50	0.82	0.60	5.30
Basophill percentage	0.84	0.65	4.48	0.79	0.59	4.77
Townsend deprivation index at recruitment	0.95	0.67	15.63	0.67	0.52	11.34
Weight	1.23	0.54	45.05	0.37	0.28	4.81
White blood cell leukocyte count	0.69	0.52	13.43	0.81	0.61	4.67
High light scatter reticulocyte count	0.81	0.61	19.22	0.62	0.48	3.31

S17 Table: Magnitude of relative change of non-BMI quantitative traits between a tissue-specific subtype group of individuals for BMI and the corresponding group of BMI-matched individuals randomly selected from the population. The magnitude of BMI-matched tissue-specific relative change of a trait is calculated as the following: $\mid \frac{\text{tissue specific mean - BMI matched random mean}}{\text{population s.d.}} \mid \times 100$, where BMI-matched random mean is the trait mean computed only in the BMI-matched (with the individuals belonging to the corresponding tissue-specific subtype group of BMI) random individuals selected from the population. The tissue-specific mean is computed only in the individuals with the corresponding tissue-specific subtype of BMI. We also provide the magnitude of primary tissue-specific relative change (before BMI-matching) of each trait which is measured by: $\mid \frac{\text{tissue specific mean - remaining population mean}}{\text{population s.d.}} \mid \times 100$. These traits were found to be differentially distributed between at least one of adipose and brain specific group of individuals and the remaining population after BMI adjustment (S15 Table).
