# Supplementary material for: Leveraging eQTLs to identify individual-level tissue of interest for a complex trait
Source: PLoS Comput Biol. 2021 May 21;17(5):e1008915. doi: 10.1371/journal.pcbi.1008915 (PMC8174686; doi:10.1371/journal.pcbi.1008915)
Supplement: S18 Table — (PDF) [file pcbi.1008915.s026.pdf]

Trait	WHRadjBMI matched AS change mean	WHRadjBMI matched AS change sd	AS change primary	WHRadjBMI matched MS change mean	WHRadjBMI matched MS change sd	MS change primary
Sitting height	3.56	0.82	15.58	30.18	1.03	18.68
Standing height	2.85	0.73	21.63	30.51	0.94	14.98
Haemoglobin concentration	0.89	0.61	16.95	24.21	1.06	7.94
Haematocrit percentage	0.61	0.46	14.40	21.90	1.08	5.65
Red blood cell erythrocyte count	0.76	0.54	11.66	20.14	1.11	5.71
Weight	3.38	0.91	19.36	16.41	1.10	9.81
Creatinine enzymatic in urine	0.66	0.50	3.13	14.27	1.16	3.35
Platelet crit	0.92	0.64	7.53	12.96	1.12	7.93
Number of treatments medications taken	5.19	0.92	12.53	8.31	1.16	19.77
Platelet count	0.74	0.55	4.84	10.78	1.14	6.49
Number of self reported non cancer illnesses	6.12	0.91	11.79	5.16	1.20	15.63
Monocyte percentage	1.23	0.77	6.11	9.91	1.19	4.58
Neuroticism score	0.78	0.58	4.48	8.73	1.26	7.91
Mean corpuscular haemoglobin concentration	1.07	0.75	7.16	7.36	1.15	6.78
Red blood cell erythrocyte distribution width	3.14	0.92	11.09	4.83	1.15	10.08
Monocyte count	0.72	0.52	2.36	6.96	1.24	3.28
Body mass index	6.07	1.00	39.22	0.97	0.72	21.86
Townsend deprivation index at recruitment	2.32	0.84	5.76	4.52	1.24	10.87
Immature reticulocyte fraction	2.44	0.92	8.25	3.81	1.19	12.68
Waist circumference	4.10	0.85	3.68	1.86	0.94	31.76
Lymphocyte count	1.81	0.78	2.59	3.96	1.25	4.58
Age completed full time education	2.17	1.06	3.10	3.35	1.35	7.13
Mean corpuscular haemoglobin	0.72	0.57	7.16	4.76	1.17	2.91
High light scatter reticulocyte percentage	1.21	0.61	3.42	4.03	1.07	10.43
White blood cell leukocyte count	1.84	0.81	5.10	2.83	1.19	11.61
Basophill count	0.75	0.56	2.28	3.71	1.21	5.58
Reticulocyte percentage	0.76	0.55	1.30	3.25	1.12	8.64
Neutrophill count	1.39	0.79	5.68	2.31	1.12	12.25
Non cancer illness code self reported	1.57	0.88	1.46	1.07	0.83	2.15
High light scatter reticulocyte count	1.60	0.82	3.64	0.97	0.69	11.99
Non cancer illness year age first occurred	0.82	0.59	1.45	1.24	0.90	7.83
Reticulocyte count	0.72	0.54	0.24	1.16	0.82	6.86

S18 Table: Magnitude of relative change of non-WHR quantitative traits between a tissue-specific subtype group of individuals for WHRadjBMI and the corresponding group of WHRadjBMI-matched individuals randomly selected from the population. The magnitude of WHRadjBMI-matched tissue-specific relative change of a trait is calculated as the following: $\left| \frac{\text{tissue specific mean - WHRadjBMI matched random mean}}{\text{population s.d.}} \right| \times 100$, where WHRadjBMI-matched random mean is the trait mean computed only in the WHRadjBMI-matched (with the individuals belonging to the corresponding tissue-specific subtype group of WHRadjBMI) random individuals selected from the population. The tissue-specific mean is computed only in the individuals with the corresponding tissue-specific subtype of WHRadjBMI. We also provide the magnitude of primary tissue-specific relative change (before WHRadjBMI-matching) of each trait which is measured by: $\left| \frac{\text{tissue specific mean - remaining population mean}}{\text{population s.d.}} \right| \times 100$. These traits were found to be differentially distributed between at least one of adipose subcutaneous (AS) and muscle skeletal (MS) specific group of individuals and the remaining population after WHRadjBMI adjustment (S16 Table).
