# Supplementary material for: Leveraging eQTLs to identify individual-level tissue of interest for a complex trait
Source: PLoS Comput Biol. 2021 May 21;17(5):e1008915. doi: 10.1371/journal.pcbi.1008915 (PMC8174686; doi:10.1371/journal.pcbi.1008915)
Supplement: S19 Table — (PDF) [file pcbi.1008915.s027.pdf]

Trait	real adip mean	shuffled adip mean sd	adipose P	real brain mean	shuffled brain mean sd	brain P	popln mean
Body mass index	30.39	29.4(1.102)	6.25E-142	27.60	28.58(1.133)	9.76E-130	27.40
Waist circumference	95.50	93.25(2.455)	1.00E-146	89.20	91.36(2.51)	2.53E-128	90.33
Weight	85.20	82.53(2.982)	4.36E-140	77.58	80.27(3.059)	2.32E-135	78.31
Standing height	167.45	167.57(0.144)	1.81E-127	167.65	167.62(0.152)	1.41E-08	168.84
Sitting height	88.97	88.95(0.054)	1.58E-32	88.82	88.89(0.054)	0	89.41
Haemoglobin concentration	14.04	14.03(0.029)	1.86E-62	13.97	13.99(0.028)	5.19E-112	14.21
Haematocrit percentage	40.76	40.72(0.083)	1.42E-60	40.54	40.63(0.079)	6.79E-227	41.16
Red blood cell erythrocyte count	4.48	4.47(0.016)	1.67E-81	4.44	4.46(0.016)	5.71E-226	4.51
High light scatter reticulocyte percentage	0.44	0.43(0.019)	3.60E-120	0.40	0.41(0.02)	2.33E-117	0.40
Red blood cell erythrocyte distribution width	13.62	13.6(0.018)	3.32E-314	13.56	13.59(0.019)	0	13.47
White blood cell leukocyte count	7.15	7.1(0.076)	1.07E-88	6.98	7.05(0.079)	1.23E-131	6.89
High light scatter reticulocyte count	0.02	0.02(0.001)	1.16E-157	0.02	0.02(0.001)	5.81E-99	0.02
Neutrophill count	4.45	4.41(0.046)	2.68E-134	4.35	4.39(0.048)	5.00E-112	4.24
Reticulocyte count	0.06	0.06(0.002)	2.21E-172	0.06	0.06(0.002)	5.46E-56	0.06
Reticulocyte percentage	1.42	1.38(0.043)	3.86E-167	1.32	1.35(0.045)	5.01E-57	1.35
Mean corpuscular haemoglobin concentration	34.46	34.46(0.01)	1.84E-08	34.48	34.46(0.01)	0	34.54
Platelet crit	0.24	0.24(0.001)	5.28E-140	0.23	0.24(0.001)	3.18E-64	0.23
Basophill count	0.04	0.04(0.001)	9.52E-77	0.04	0.04(0.001)	1.08E-163	0.03
Monocyte percentage	6.99	6.98(0.026)	5.52E-78	6.97	6.98(0.026)	1.07E-26	7.10
Neutrophill percentage	61.67	61.68(0.077)	0.0004	61.67	61.72(0.079)	1.20E-61	61.14
Mean corpuscular volume	91.09	91.2(0.148)	3.37E-89	91.49	91.32(0.155)	1.56E-210	91.34
Platelet count	256.95	256.41(0.845)	2.72E-71	255.58	256.01(0.928)	6.14E-39	253.21
Lymphocyte percentage	28.20	28.22(0.067)	4.46E-07	28.25	28.19(0.07)	2.15E-114	28.63
Monocyte count	0.49	0.48(0.005)	7.09E-222	0.47	0.48(0.005)	3.00E-225	0.48
Basophill percentage	0.59	0.59(0.006)	3.44E-07	0.60	0.6(0.006)	0.02	0.57
Number of treatments medications taken	3.08	2.9(0.132)	0	2.70	2.81(0.135)	3.64E-120	2.45
Number of self reported non cancer illnesses	2.24	2.12(0.087)	0	1.98	2.07(0.088)	4.23E-156	1.87
Interpolated Age of participant when non cancer illness first diagnosed	41.89	41.65(0.256)	8.57E-144	41.56	41.46(0.263)	6.73E-26	42.32
Townsend deprivation index at recruitment	-1.13	-1.17(0.055)	9.18E-71	-1.26	-1.18(0.057)	1.54E-316	-1.58
Neuroticism score	4.44	4.4(0.032)	2.37E-322	4.38	4.41(0.033)	1.51E-120	4.10
Creatinine enzymatic in urine	9077.69	8885.47(252.885)	3.80E-101	8429.45	8677.5(254.514)	5.26E-165	8806.93
Sodium in urine	79.56	77.86(1.986)	7.51E-127	74.72	76.34(2.001)	1.92E-114	76.21
Sleep duration	7.08	7.08(0.014)	7.10E-21	7.08	7.08(0.014)	2.82E-26	7.13
Immature reticulocyte fraction	0.30	0.3(0.004)	2.20E-127	0.29	0.29(0.004)	4.98E-141	0.29
Mean corpuscular haemoglobin	31.39	31.43(0.052)	7.04E-99	31.55	31.46(0.055)	0	31.55
Lymphocyte count	1.98	1.98(0.024)	1.06E-16	1.93	1.96(0.025)	1.32E-179	1.95
Eosinophill count	0.18	0.18(0.003)	3.41E-43	0.17	0.18(0.003)	6.94E-66	0.17
Non cancer illness code self reported	2405.55	2696.21(140.196)	0	2779.19	2747.36(146.249)	9.77E-10	2891.97
Non cancer illness year age first occurred	628.73	646.05(28.147)	6.08E-67	703.82	665.47(29.619)	1.36E-289	702.47
Mean sphered cell volume	82.86	83(0.201)	3.20E-90	83.37	83.18(0.206)	1.21E-147	82.87
Mean reticulocyte volume	106.00	106.02(0.125)	3.58E-05	106.22	106.13(0.13)	3.50E-83	105.83
Eosinophill percentage	2.53	2.53(0.018)	6.55E-10	2.52	2.52(0.019)	0.71	2.56
Platelet distribution width	16.49	16.48(0.007)	0	16.47	16.48(0.007)	2.24E-205	16.49
Potassium in urine	63.61	62.84(0.851)	1.64E-141	61.68	62.13(0.858)	8.05E-48	63.47
Birth weight	3.30	3.3(0.009)	0.0001	3.30	3.3(0.009)	8.54E-06	3.33

S19 Table: Summary of results from the analyses performed to characterize the tissue-specificity of phenotypic characteristics of individuals assigned to a tissue-specific subtype of BMI. Central tendency measures of the artificial tissue-specific mean of a quantitative trait (computed only in the individuals classified into artificial tissue-specific subtype of BMI) across 500 artificial tissue-specific subtype groups identified based on 500 sets of artificial tissue-specific eQTLs (obtained by random exchange of eQTLs between the sets of adipose and brain specific eQTLs) are provided. These quantitative traits were found primarily heterogeneous between at least one of real adipose and brain specific subtype groups of individuals and the remaining population (S10 Table). Here adipose P denotes the p-value of testing whether the artificial adipose tissue-specific trait mean across the artificial adipose tissue-specific subtype groups is different from the original adipose tissue-specific trait mean. Similarly Brain P is defined. The real adipose and brain tissue-specific mean and population mean of the traits are also provided.
