# Supplementary material for: Leveraging eQTLs to identify individual-level tissue of interest for a complex trait
Source: PLoS Comput Biol. 2021 May 21;17(5):e1008915. doi: 10.1371/journal.pcbi.1008915 (PMC8174686; doi:10.1371/journal.pcbi.1008915)
Supplement: S20 Table — (PDF) [file pcbi.1008915.s028.pdf]

Trait	real AS mean	shuffled AS mean sd	AS P	real MS mean	shuffled MS mean sd	MS P	popln mean
WHRadjBMI	-0.04	-0.01(0.018)	0	0.03	-0.01(0.018)	0	0.00
WHR	0.85	0.88(0.017)	0	0.91	0.88(0.017)	0	0.87
Body mass index	29.19	28.81(0.232)	0	28.41	28.76(0.223)	0	27.39
Standing height	166.91	167.14(0.152)	0	167.48	167.17(0.15)	0	168.84
Sitting height	88.68	88.6(0.105)	7.55E-117	88.53	88.62(0.102)	2.92E-129	89.41
Weight	81.27	80.53(0.513)	0	79.83	80.42(0.488)	2.76E-258	78.31
Haemoglobin concentration	14.01	14.06(0.029)	0	14.12	14.06(0.028)	0	14.21
Haematocrit percentage	40.67	40.78(0.075)	0	40.96	40.78(0.074)	0	41.16
Red blood cell erythrocyte distribution width	13.57	13.56(0.012)	4.15E-198	13.56	13.55(0.011)	2.64E-86	13.47
Red blood cell erythrocyte count	4.47	4.48(0.007)	2.12E-289	4.49	4.48(0.007)	0	4.51
High light scatter reticulocyte percentage	0.41	0.42(0.007)	0	0.43	0.42(0.007)	0	0.40
White blood cell leukocyte count	6.99	7.05(0.058)	1.29E-220	7.12	7.04(0.057)	2.30E-300	6.89
Immature reticulocyte fraction	0.29	0.3(0.001)	1.31E-202	0.30	0.3(0.001)	1.62E-205	0.29
Neutrophill count	4.32	4.37(0.039)	7.42E-258	4.41	4.36(0.038)	7.97E-298	4.24
Mean corpuscular haemoglobin concentration	34.47	34.48(0.014)	3.47E-139	34.47	34.48(0.013)	8.32E-125	34.54
Monocyte percentage	6.94	6.98(0.031)	1.34E-285	6.98	6.99(0.03)	3.47E-08	7.10
Platelet crit	0.24	0.24(0.001)	1.54E-98	0.24	0.24(0.001)	3.98E-12	0.23
Lymphocyte count	1.98	1.98(0.017)	6.63E-15	2.00	1.98(0.016)	1.18E-259	1.95
Platelet count	256.00	257.04(0.987)	8.71E-192	257.00	256.89(0.957)	0.001	253.21
Number of treatments medications taken	2.77	2.86(0.068)	1.89E-288	2.97	2.85(0.067)	0	2.45
Number of self reported non cancer illnesses	2.08	2.12(0.045)	4.48E-154	2.15	2.11(0.044)	1.61E-145	1.86
Townsend deprivation index at recruitment	-1.41	-1.33(0.06)	0	-1.26	-1.34(0.059)	4.50E-285	-1.58
Neuroticism score	4.24	4.31(0.058)	1.24E-223	4.35	4.3(0.056)	7.90E-147	4.10
Mean corpuscular haemoglobin	31.43	31.46(0.026)	0	31.50	31.47(0.026)	4.64E-271	31.55
Monocyte count	0.47	0.48(0.004)	0	0.49	0.48(0.005)	4.75E-203	0.48
Mean corpuscular volume	91.18	91.25(0.056)	3.11E-257	91.38	91.26(0.055)	0	91.34
Mean platelet thrombocyte volume	9.36	9.35(0.016)	6.32E-226	9.33	9.35(0.016)	5.81E-136	9.32
Eosinophill percentage	2.49	2.52(0.018)	0	2.53	2.52(0.019)	3.38E-49	2.56
Creatinine enzymatic in urine	8633.55	8672.89(66.169)	1.54E-62	8618.76	8670.39(67.216)	4.07E-103	8806.64
Waist circumference	90.80	92.5(0.98)	0	94.50	92.39(0.981)	0	90.33
High light scatter reticulocyte count	0.02	0.02(0)	0	0.02	0.02(0)	0	0.02
Reticulocyte percentage	1.36	1.39(0.019)	0	1.42	1.38(0.019)	0	1.35
Reticulocyte count	0.06	0.06(0.001)	0	0.06	0.06(0.001)	0	0.06
Basophill count	0.03	0.04(0.001)	4.27E-248	0.04	0.04(0.001)	0	0.03
Non cancer illness code self reported	3071.24	2746.71(158.474)	0	2623.36	2747.26(158.328)	5.81E-107	2891.68
Non cancer illness year age first occurred	689.59	655.07(14.516)	0	631.65	655.89(14.155)	0	702.50
Age completed full time education	16.36	16.32(0.044)	3.26E-150	16.25	16.33(0.042)	0	16.45

S20 Table: Summary of results from the analyses performed to characterize the tissue-specificity of phenotypic characteristics of the individuals assigned to a tissue-specific subtype of WHRadjBMI. Central tendency measures of the artificial tissue-specific mean of a quantitative trait (computed only in the individuals classified into artificial tissue-specific subtype of WHRadjBMI) across 500 artificial tissue-specific subtype groups identified based on 500 sets of artificial tissue-specific eQTLs (obtained by random exchange of eQTLs between the sets of adipose subcutaneous (AS) and muscle skeletal (MS) specific eQTLs) are provided. These quantitative traits were found primarily heterogeneous between at least one of real AS and MS specific subtype groups of individuals and the remaining population (S11 Table). Here AS P denotes the p-value of testing whether the artificial AS tissue-specific trait mean across the artificial AS tissue-specific subtype groups is different from the original AS tissue-specific trait mean. Similarly MS P is defined. The real AS and MS tissue-specific mean and overall population mean of the traits are also provided.
