# Supplementary material for: Leveraging eQTLs to identify individual-level tissue of interest for a complex trait
Source: PLoS Comput Biol. 2021 May 21;17(5):e1008915. doi: 10.1371/journal.pcbi.1008915 (PMC8174686; doi:10.1371/journal.pcbi.1008915)
Supplement: S21 Table — (PDF) [file pcbi.1008915.s029.pdf]

Trait	real adip mean	permuted adip mean sd	adip P	real brain mean	permuted brain mean sd	brain P	popln mean
Body mass index	30.39	29.8(2.579)	1.01E-09	27.60	30.08(2.569)	1.19E-143	27.40
Waist circumference	95.50	93.33(5.564)	4.98E-25	89.20	93.92(5.526)	3.68E-113	90.33
Weight	85.20	83.11(6.898)	8.55E-16	77.58	83.85(6.867)	3.00E-129	78.31
Standing height	167.45	167.2(0.354)	2.63E-76	167.65	167.15(0.358)	3.09E-289	168.84
Sitting height	88.97	88.76(0.104)	0	88.82	88.76(0.104)	3.62E-41	89.41
Haemoglobin concentration	14.04	13.94(0.05)	0	13.97	13.94(0.049)	2.55E-76	14.21
Haematocrit percentage	40.76	40.51(0.146)	0	40.54	40.51(0.146)	2.05E-06	41.16
Red blood cell erythrocyte count	4.48	4.45(0.032)	2.04E-179	4.44	4.45(0.031)	2.60E-21	4.51
High light scatter reticulocyte percentage	0.44	0.43(0.042)	1.60E-24	0.40	0.43(0.042)	1.82E-102	0.40
Red blood cell erythrocyte distribution width	13.62	13.66(0.045)	7.23E-120	13.56	13.67(0.045)	0	13.47
White blood cell leukocyte count	7.15	7.16(0.174)	0.23	6.98	7.18(0.171)	4.52E-212	6.89
High light scatter reticulocyte count	0.02	0.02(0.002)	1.17E-37	0.02	0.02(0.002)	1.27E-91	0.02
Neutrophill count	4.45	4.48(0.109)	1.42E-11	4.35	4.49(0.106)	5.09E-297	4.24
Reticulocyte count	0.06	0.06(0.005)	1.36E-71	0.06	0.06(0.005)	4.74E-45	0.06
Reticulocyte percentage	1.42	1.37(0.095)	1.56E-59	1.32	1.37(0.093)	1.05E-50	1.35
Mean corpuscular haemoglobin concentration	34.46	34.42(0.021)	0	34.48	34.41(0.02)	0	34.54
Platelet crit	0.24	0.24(0.002)	0.6	0.23	0.24(0.002)	6.25E-167	0.23
Basophill count	0.04	0.04(0.001)	9.23E-126	0.04	0.04(0.001)	0	0.03
Monocyte percentage	6.99	6.93(0.06)	1.89E-181	6.97	6.92(0.062)	2.94E-83	7.10
Neutrophill percentage	61.67	62.08(0.16)	0	61.67	62.09(0.161)	0	61.14
Mean corpuscular volume	91.09	91.23(0.347)	5.10E-24	91.49	91.2(0.345)	1.78E-108	91.34
Platelet count	256.95	257.38(1.965)	5.57E-09	255.58	257.64(1.987)	2.58E-166	253.21
Lymphocyte percentage	28.20	27.88(0.139)	0	28.25	27.87(0.144)	0	28.63
Monocyte count	0.49	0.48(0.011)	3.15E-47	0.47	0.48(0.011)	1.61E-156	0.48
Basophill percentage	0.59	0.61(0.012)	2.86E-240	0.60	0.61(0.013)	1.49E-134	0.57
Number of treatments medications taken	3.08	3.03(0.3)	8.80E-06	2.70	3.07(0.301)	7.88E-229	2.45
Number of self reported non cancer illnesses	2.24	2.2(0.192)	5.35E-11	1.98	2.22(0.19)	8.09E-234	1.87
Interpolated Age of participant when non cancer illness first diagnosed	41.89	41.2(0.503)	1.70E-281	41.56	41.22(0.502)	8.36E-71	42.32
Townsend deprivation index at recruitment	-1.13	-0.98(0.12)	6.98E-266	-1.26	-0.96(0.119)	0	-1.58
Neuroticism score	4.44	4.55(0.065)	0	4.38	4.55(0.067)	0	4.10
Creatinine enzymatic in urine	9077.69	8794.61(534.421)	1.27E-44	8429.45	8846.33(529.198)	1.79E-96	8806.93
Sodium in urine	79.56	77.5(4.269)	3.10E-37	74.72	77.98(4.235)	9.31E-92	76.21
Immature reticulocyte fraction	0.30	0.3(0.01)	6.63E-17	0.29	0.3(0.009)	1.19E-129	0.29
Mean corpuscular haemoglobin	31.39	31.4(0.125)	0.04	31.55	31.39(0.126)	4.92E-258	31.55
Lymphocyte count	1.98	1.97(0.052)	2.20E-18	1.93	1.97(0.052)	8.81E-77	1.95
Eosinophill count	0.18	0.18(0.006)	9.52E-07	0.17	0.18(0.007)	1.51E-78	0.17
Non cancer illness code self reported	2405.55	2629.07(294.182)	6.95E-90	2779.19	2607.87(286.357)	1.98E-56	2891.97
Non cancer illness year age first occurred	628.73	642.48(62.848)	7.07E-09	703.82	635.71(61.989)	8.73E-186	702.47
Mean sphered cell volume	82.86	83.21(0.444)	5.65E-98	83.37	83.18(0.444)	3.20E-29	82.87
Mean reticulocyte volume	106.00	106.24(0.27)	7.31E-127	106.22	106.23(0.263)	0.06	105.83
Eosinophill percentage	2.53	2.51(0.04)	4.40E-55	2.52	2.51(0.042)	0.003	2.56
Platelet distribution width	16.49	16.48(0.013)	8.96E-244	16.47	16.48(0.013)	1.06E-54	16.49
Potassium in urine	63.61	62.16(1.764)	3.31E-105	61.68	62.29(1.727)	1.30E-20	63.47
Birth weight	3.30	3.29(0.018)	3.09E-83	3.30	3.29(0.018)	1.67E-25	3.33

S21 Table: Summary of results on the phenotypic characteristics of the individuals assigned to tissue-specific subtype of BMI identified based on random permutations of the primary phenotype (BMI) across individuals. Central tendency measures of the tissue-specific mean of a quantitative trait (computed only in the individuals classified into tissue-specific subtype detected based on a randomly permuted BMI data) across 500 tissue-specific subtype groups identified based on 500 random permutations of BMI data are provided. These quantitative traits were found primarily heterogeneous between at least one of real adipose and brain specific subtype groups of individuals and the remaining population (S10 Table). Here adipose P denotes the p-value of testing whether a trait mean computed only in the random adipose subtype group of individuals (detected based on random permutations of BMI) is different from the original adipose tissue-specific trait mean. Similarly Brain P is defined. The real adipose and brain tissue-specific mean and overall population mean of the traits are also provided.
