# Supplementary material for: Leveraging eQTLs to identify individual-level tissue of interest for a complex trait
Source: PLoS Comput Biol. 2021 May 21;17(5):e1008915. doi: 10.1371/journal.pcbi.1008915 (PMC8174686; doi:10.1371/journal.pcbi.1008915)
Supplement: S22 Table — (PDF) [file pcbi.1008915.s030.pdf]

Trait	real AS mean	permuted AS mean sd	AS P	real MS mean	permuted MS mean sd	MS P	popln mean
WHRadjBMI	-0.04	0(0.002)	0	0.03	0(0.002)	0	0.00
WHR	0.85	0.87(0.003)	0	0.91	0.87(0.002)	0	0.87
Body mass index	29.19	27.39(0.126)	0	28.41	27.38(0.126)	0	27.39
Standing height	166.91	168.83(0.265)	0	167.48	168.84(0.241)	0	168.84
Sitting height	88.68	89.41(0.143)	0	88.53	89.41(0.129)	0	89.41
Weight	81.27	78.3(0.428)	0	79.83	78.28(0.415)	0	78.31
Haemoglobin concentration	14.01	14.21(0.034)	0	14.12	14.21(0.033)	0	14.21
Haematocrit percentage	40.67	41.15(0.098)	0	40.96	41.15(0.094)	0	41.16
Red blood cell erythrocyte distribution width	13.57	13.47(0.026)	0	13.56	13.47(0.025)	0	13.47
Red blood cell erythrocyte count	4.47	4.51(0.011)	0	4.49	4.51(0.011)	0	4.51
High light scatter reticulocyte percentage	0.41	0.4(0.007)	0	0.43	0.4(0.009)	0	0.40
White blood cell leukocyte count	6.99	6.89(0.057)	0	7.12	6.88(0.054)	0	6.89
Immature reticulocyte fraction	0.29	0.29(0.002)	0	0.30	0.29(0.002)	0	0.29
Neutrophill count	4.32	4.24(0.039)	0	4.41	4.24(0.038)	0	4.24
Mean corpuscular haemoglobin concentration	34.47	34.54(0.029)	0	34.47	34.54(0.029)	0	34.54
Monocyte percentage	6.94	7.1(0.073)	0	6.98	7.1(0.073)	0	7.10
Platelet crit	0.24	0.23(0.001)	0	0.24	0.23(0.001)	0	0.23
Lymphocyte count	1.98	1.95(0.034)	3.75E-101	2.00	1.95(0.031)	0	1.95
Platelet count	256.00	253.25(1.673)	0	257.00	253.16(1.545)	0	253.21
Number of treatments medications taken	2.77	2.46(0.066)	0	2.97	2.45(0.071)	0	2.45
Number of self reported non cancer illnesses	2.08	1.86(0.047)	0	2.15	1.87(0.05)	0	1.86
Townsend deprivation index at recruitment	-1.41	-1.57(0.081)	0	-1.26	-1.58(0.074)	0	-1.58
Neuroticism score	4.24	4.11(0.099)	1.62E-288	4.35	4.1(0.095)	0	4.10
Mean corpuscular haemoglobin	31.43	31.55(0.05)	0	31.50	31.55(0.049)	2.33E-187	31.55
Monocyte count	0.47	0.48(0.006)	8.74E-115	0.49	0.48(0.006)	1.64E-225	0.48
Mean corpuscular volume	91.18	91.34(0.119)	1.68E-247	91.38	91.35(0.113)	7.97E-19	91.34
Mean platelet thrombocyte volume	9.36	9.32(0.029)	5.66E-313	9.33	9.32(0.03)	1.90E-17	9.32
Eosinophill percentage	2.49	2.56(0.049)	5.9E-318	2.53	2.56(0.047)	4.76E-39	2.56
Creatinine enzymatic in urine	8633.55	8797.42(156.859)	3.61E-168	8618.76	8808.86(151.926)	2.52E-240	8806.64
Waist circumference	90.80	90.32(0.367)	1.52E-269	94.50	90.3(0.355)	0	90.33
High light scatter reticulocyte count	0.02	0.02(0)	7.67E-277	0.02	0.02(0)	0	0.02
Reticulocyte percentage	1.36	1.35(0.023)	8.63E-42	1.42	1.35(0.023)	0	1.35
Reticulocyte count	0.06	0.06(0.001)	0.006	0.06	0.06(0.001)	0	0.06
Basophill count	0.03	0.03(0.002)	9.45E-88	0.04	0.03(0.001)	0	0.03
Non cancer illness code self reported	3071.24	2887.54(384.658)	1.35E-36	2623.36	2869.04(391.165)	5.20E-62	2891.68
Non cancer illness year age first occurred	689.59	702.72(28.466)	2.93E-34	631.65	702.56(28.388)	0	702.50
Age completed full time education	16.36	16.46(0.092)	1.10E-155	16.25	16.44(0.093)	0	16.45

S22 Table: Summary of results on the phenotypic characteristics of the individuals assigned to tissue-specific subtype of WHRadjBMI identified based on random permutations of the primary phenotype (WHRadjBMI) across individuals. Central tendency measures of the tissue-specific mean of a quantitative trait (computed only in the individuals classified into a tissue-specific subtype detected based on a randomly permuted WHRadjBMI data) across 500 tissue-specific subtype groups identified based on 500 random permutations of WHRadjBMI data are provided. These quantitative traits were found primarily heterogeneous between at least one of real adipose subcutaneous (AS) and muscle skeletal (MS) specific subtype groups of individuals and the remaining population (S11 Table). Here AS P denotes the p-value of testing whether a trait mean computed only in the random AS-specific subtype group of individuals (detected based on random permutations of WHRadjBMI) is different from the original AS tissue-specific trait mean. Similarly MS P is defined. The real AS and MS tissue-specific mean and overall population mean of the traits are also provided.
