# Supplementary material for: Leveraging eQTLs to identify individual-level tissue of interest for a complex trait
Source: PLoS Comput Biol. 2021 May 21;17(5):e1008915. doi: 10.1371/journal.pcbi.1008915 (PMC8174686; doi:10.1371/journal.pcbi.1008915)
Supplement: S23 Table — (PDF) [file pcbi.1008915.s031.pdf]

Trait	P value	Mean		s.d.	
		Adipose	Brain	Adipose	Brain
Weight	5.45E-137	85.20	77.57	25.02	25.18
Waist circumference	9.36E-137	95.51	89.20	20.25	20.57
Body mass index	1.08E-127	30.39	27.60	8.60	8.74
High light scatter reticulocyte count	2.73E-73	0.02	0.02	0.01	0.01
Reticulocyte count	2.63E-68	0.06	0.06	0.04	0.05
High light scatter reticulocyte percentage	2.25E-66	0.44	0.40	0.25	0.27
Reticulocyte percentage	3.34E-60	1.42	1.32	0.84	1.05
Immature reticulocyte fraction	1.75E-37	0.30	0.29	0.07	0.07
Number of self reported non cancer illnesses	1.47E-26	2.25	1.98	2.15	2.02
Number of treatments medications taken	2.65E-26	3.08	2.70	3.16	2.96
Creatinine enzymatic in urine	2.87E-22	9075.79	8432.19	5935.93	5815.15
Red blood cell erythrocyte count	2.67E-18	4.48	4.44	0.41	0.41
Sodium in urine	5.76E-18	79.58	74.73	45.87	44.42
White blood cell leukocyte count	1.34E-15	7.15	6.98	1.96	2.01
Non cancer illness code self reported	2.89E-14	2404.66	2779.67	10879.75	12369.88
Mean sphered cell volume	3.46E-14	82.85	83.37	5.55	5.59
Mean corpuscular volume	1.48E-13	91.09	91.49	4.77	4.65
Mean corpuscular haemoglobin	9.72E-13	31.39	31.55	1.93	2.03
Neutrophill count	9.35E-11	4.45	4.35	1.52	1.52
Lymphocyte count	5.73E-10	1.98	1.93	0.76	0.71
Monocyte count	2.10E-09	0.49	0.47	0.24	0.20
Haematocrit percentage	1.86E-08	40.77	40.54	3.53	3.44
Potassium in urine	4.56E-08	63.59	61.69	33.46	33.87
Haemoglobin concentration	8.49E-08	14.04	13.97	1.23	1.19
Eosinophill count	6.61E-07	0.18	0.17	0.13	0.14
Red blood cell erythrocyte distribution width	1.01E-06	13.62	13.56	1.07	1.03
Townsend deprivation index at recruitment	0.0003	-1.13	-1.26	3.12	3.12

S23 Table: Quantitative traits among 106 phenotypes in UK Biobank which are differentially distributed between the adipose-specific subtype group of individuals for BMI and brain-specific subtype group of individuals. We provide the p-values of testing heterogeneity of each trait between the tissue-specific subtype groups. For each trait, the tissue-specific (adipose and brain) mean and standard deviation which are calculated only in the individuals classified as the corresponding tissue-specific subtype of BMI are provided.
