# Supplementary material for: Leveraging eQTLs to identify individual-level tissue of interest for a complex trait
Source: PLoS Comput Biol. 2021 May 21;17(5):e1008915. doi: 10.1371/journal.pcbi.1008915 (PMC8174686; doi:10.1371/journal.pcbi.1008915)
Supplement: Attachment — Submitted filename: reviewrs-response.pdf [file pcbi.1008915.s032.pdf]

Letter of response to reviewers comments for Majumdar et al *“Leveraging eQTLs to identify individual-level tissue of interest for a complex trait”*

We appreciate the constructive feedback from the reviewers that have allowed us to greatly improve the quality of our manuscript. You will find below a point-by-point response to each of the reviewer comments together with details on how the revised manuscript addresses all suggestions. For ease of presentation we highlight all our answers and changes in the main manuscript in blue fonts.

Kind regards,

Arunabha Majumdar (on behalf of all co-authors)

Reviewer 1:

In this manuscript, Majumdar et al. propose a novel method to quantify the tissue-wise genetic contribution to the trait of interest at the individual level. Briefly, they use a mixture model, which integrates tissue-specific eQTLs with genetic association data, to identify subgroups of individuals whose genetic predisposition act primarily through one specific trait. They showcase the utility of their method by extensive simulations and real data analyses of UK Biobank data. I find the manuscript is very interesting and may further help us understand the etiology of complex traits. However, I have a few questions/comments that hopefully the authors can consider to address.

Response: We thank the reviewer for the kind words.

Major comments

1. *A 65% threshold of tissue-specific subtype posterior probability was used. Is there any justification for this threshold? Are the results (especially those in real data analyses) sensitive to the choice of threshold?*

   Response: We thank the reviewer for the interesting suggestion. Indeed, in the initial revision we focused on the 65% threshold as that achieved a good balance in the number of classifications (~ 19,000 – 25,000 individuals classified at this threshold) and accuracy. As noted by the reviewer and as expected at more stringent thresholds (e.g., 70%) a smaller number of individuals are classified in the components.

   Motivated by reviewers suggestion, We now repeat the analysis of phenotypic characteristics of tissue-specific subtype groups for BMI based on 70% threshold of posterior probability. We observe a similar pattern of phenotypic heterogeneity between a tissue-specific subtype group and the remaining population as before (using 65% threshold). Using the 70% threshold we found 87 phenotypes to be heterogeneously distributed compared to 85 phenotypes obtained by using the 65% threshold. The sets of heterogeneous phenotypes are highly overlapping between the two choices. Thus, results on phenotypic heterogeneity were not sensitive to one of these two choices. For brevity, we skip providing detailed results on phenotypic heterogeneity obtained by using the 70% threshold. We note that if we consider a much higher choice of the posterior probability threshold, the size of the tissue-specific subtype group will be small. In such a scenario, even though the identified subtype groups will have fewer misclassified individuals, the statistical power to identify heterogeneously distributed phenotypes would decrease mainly due to substantially lower sample size of the subtype groups. For example, using 70%, 80%, 90% thresholds of posterior probability in the BMI analysis, the number of classified individuals were 11871, 2085, 177, respectively. We now elaborately discuss this point in the discussion section on page 10, lines 379 - 397.

2. *How the tissue-specific genes and eQTL are selected? What's the specific cutoff we are using (on Line 583)? I am just curious why "we included the top eQTL of the gene in both tissues, one in subcutaneous and one in visceral." Is there any consideration for that? Why not just simply exclude those genes to make the eQTL lists more tissue-specific?*

   Response: We adopted the list of tissue-specific expressed genes provided by Finucane et al. (Nature Genetics, 2018) for our analysis. Based on the expression data in GTEx, Finucane et al. considered a gene to be specifically expressed in a tissue if the gene's mean expression in the tissue is substantially higher than its mean expression in other tissues combined, and calculated a t-statistic to rank the genes with respect to higher expression in a specific tissue. Following their work, we considered the top 10% of the genes expressed in a tissue, ranked according to descending value of the tissue-specific t-statistic, as the set of genes specifically expressed in the tissue. A gene is considered to be an eGene if there is at least one cis-eQTL significantly associated with its expression at FDR level 0.05 (GTEx consortium, Science, 2015). For each tissue-specific expressed eGene, we considered the top eQTL for the gene (the cis-eQTL which has the strongest p-value of association with the gene's expression). We now add the information on how an eGene is defined on page 19, line 689.

   In BMI analysis, we merged adipose subcutaneous and adipose visceral tissues to represent a unified adipose tissue. If a gene is both adipose subcutaneous and visceral specific, that is, it is relatively overexpressed in both types of adipose tissue, in overall, it is also over-expressed in the adipose tissue. Since in the BMI analysis, we considered an overall adipose tissue instead of the particular type of adipose tissue, we included these genes in the adipose-specific set. For such a gene, if the top eQTL in the two types of adipose tissues are different, these two eQTLs are also overall adipose-specific. We note that we finally LD-filtered the adipose-specific set of eQTLs. Even though the number of common genes overexpressed in both types of adipose tissue was small, removing such genes would finally reduce the total number of adipose-specific eQTLs, which would lead to a lower adipose-specific heritability, and hence discovery of a smaller number of individuals with a tissue-specific subtype.

Minor comments:

1. *On Line 121, does the sigma_{y_k}^{2} is the same no matter C_i = 1 or C_i = 2? The trait variance seems the same as we deal with the same trait.*

   Response: Here, k is the index for the tissue-specific subtype of the trait. So, for k^th tissue, k=1,2, sigma_{y_k}^{2} denotes the variance of the phenotype for the individuals with C_i=k. Even though the overall variance of the phenotype is fixed, the variance of the phenotype for the individuals with one tissue-specific subtype can be different from the variance of the phenotype for the individuals having the other tissue-specific subtype. Thus, in the mixture distribution, we considered the variance term to be

dependent on the status of C_i. We note that this is more general and also includes the specific case of the same phenotypic variance across subtypes: *sigma_{y_1}^{2}* = *sigma_{y_2}^{2}.*

2. *On line 468, some explanations regarding why using 5% are appreciated.*

   Response: Since the variance explained for a quantitative trait should be higher than that for a case-control trait, we can expect that quantitative traits have genome-wide SNP-heritability more than 5%. Here, we focus on tissue-specific eQTLs only. Hence, in the prior, we considered a conservative choice of the expected tissue-specific subtype heritability. Since this is a heuristic choice, we experimented with other choices of this prior quantity as well. Since the sample size is large in contemporary GWAS, a bit of variation in this prior choice has negligible effect on the final results. We now clarify this point in the discussion section on page 9, lines 371-378.

3. *One line 432, I feel that starting with Model 2 may be much easier to follow and understand. Model (1) seems misleading, especially if we read the Results section first. It is just a minor suggestion. It's Okay to keep the current form that starts with a more general model.*

   Response: Yes, model 1 is more general and model 2 is deduced from model 1 based on the proposed hypothesis. This is the main reason why we placed the model 1 first. For ease in understanding, we have now made a separate paragraph containing model 2.

Reviewer 2:

In this paper, the authors describe a statistical framework to infer the causal tissue per-individual underlying the predisposition for a specific complex trait. One of the applications of this is to determine subtypes of a complex trait and assign individuals to it. While the aim of the study and the proposed methods are of interest, I do however have some comments/questions/concerns:

Response: We thank the reviewer for the constructive feedback.

1. *I would be curious to see what happens when using at least one irrelevant tissue (determined from biological knowledge or common sense). All the work described here starts from tissues that were previously inferred as relevant to the studied complex trait. What happens to the classification when one of the tissues being used is known to be independent of the trait? Are there any individuals being assigned to this dummy tissue?*

   Response: This is an excellent point. For a complex trait, we chose the tissues which were reported to be significantly relevant for the trait by previous studies. For example, Finucane et al. (Nat. Genet., 2018) and Ongen et al. (Nat. Genet., 2017) proposed

statistical approaches to identify the tissues relevant for a complex trait. However, limited sample size of expression datasets (e.g., GTEx has limited sample size across different tissues) affect the power of such methods. Thus, a tissue which is not identified to be significantly relevant for the phenotype may not be completely irrelevant for the phenotype. Therefore, using real data, it may be challenging to explore the performance of eGST when an irrelevant tissue is included in the model, because it is challenging to include a tissue perfectly irrelevant for the trait. However, we ran the model with brain tissue and sun exposed skin in the lower leg. The latter tissue was replaced in place of adipose. We observe that a large number of individuals were still assigned to skin tissue based on a 65% threshold of tissue-specific posterior probability. The number of individuals assigned to skin (9,576) was substantially smaller compared to the number of individuals assigned to adipose in the primary analysis considering brain and adipose. Also, the number of individuals assigned to brain (10,785) was larger than that for skin. We observed in our previous analysis that every tissue-specific set of eQTLs has a small but positive heritability for the complex trait, which would lead to assignment of some individuals to the tissue which seems irrelevant for the trait from a general biological point of view.

Since simulated data is a cleaner option to investigate this hypothesis, we performed simulations to explore this question. We simulated phenotype data where all individuals originally had genetic effect from one tissue-specific set of SNPs, and then ran eGST including an irrelevant tissue-specific set of SNPs in the model. We observe that a very small percentage of individuals were misclassified to the dummy tissue, and the percentage of correctly classified individuals was much larger. For example, when the heritability of the trait due to the relevant tissue-specific set of SNPs is 20\%, the percentage of correctly classified individuals is 89% whereas the percentage of misclassified individuals is 1.4%. We also observe that the percentage of misclassification decreases as the choice of the threshold of the tissue-specific subtype posterior probability increases. We now include these simulation results in the supplementary material (Table S7), and discuss the results in the simulation section on page 6, lines 191-202.

2. *I understand that using genes specifically expressed for each tissue helps to avoid any ambiguities. However, GTEx introduced multiple methods and metrics to pinpoint causal eQTL variants and therefore accurately determine that a eQLT-eGene pair is specific to a given tissue. Can this information be used to improve the assignment (proportion & accuracy) of individuals to subtypes? If yes, to which extent?*

Response: We thank the reviewer for raising this interesting insight. Kitsak et al. (2016, scientific reports) have pointed out that a promising strategy to comprehend tissue-specificity underlying a complex phenotype is to consider the genes that are specifically expressed in the tissue. It will be interesting to investigate the performance of eGST when tissue-specific causal eQTLs for each eGene in the tissue are included in the model. However, the distribution of expression of a large proportion of genes may not be distinctive across tissues, and hence may not be informative of the tissue-specific

expression pattern. Thus, considering all tissue-specific eQTL,eGene pairs can increase the noise in the model while identifying the tissue-specific subtypes. However, an extensive study is needed to adequately investigate this. For simplicity in the current paper, we leave this exploration as a future work. We now discuss this point in the discussion section on page 12, lines 495-501.

3. *In UK Biobank, >100,000 samples are related at least to the 3rd degree. How does this high level of relatedness between GWAS samples affect your modeling? I understand that population stratification is accounted for thanks to PCs, but what about relatedness?*

   Response: The individuals that we used are unrelated at least to third degree relatives, i.e., a pair of individuals can only be related as fourth or higher degree relatives. Thus, cryptic relatedness remaining beyond fourth degree relatives should not impact the results substantially. However, we agree that, before running eGST, we should ideally fit a linear mixed model to adjust the phenotype for both population structure and cryptic relatedness. We now clarify this point in the discussion section, page 10, lines 398-403.

4. *For BMI and WHRadjBMI, you managed to assign a tissue to 7.5% and 5.7% individuals, respectively. I understand that some of the causes for these relatively low percentages are technical, however is there also any biological rationale behind these?*

   Response: We list a few possible biological reasons as follows: 1. a substantial proportion of individuals may have their genetic susceptibility mediated through both tissues; 2. more than two tissues can be biologically relevant for the phenotype; 3. the set of tissue-specific over expressed genes were considered to represent tissue-specificity, but an interesting possibility is to include lower-expressed genes in a tissue as well. We now discuss these points in the discussion section on page 11, lines 456-461.

5. *When permuting the phenotype data in the case of real data, you get 7,404 and 3,433 individuals being assigned a tissue. On non-permuted data, you get 25,192 and 19,041 individuals with a tissue being assigned. Am I correct to say that you have 30% and 18% false discovery rate (FDR), respectively? If yes, can you comment on this?*

   Response: We found it very challenging to interpret this result explicitly in terms of FDR. However, we agree that this result indicates that the FDR in classification is substantially large. Possible reasons include the following: 1. We used a relaxed threshold of 65% posterior probability for classification (simulation results show that the true discovery rate increases with posterior probability threshold (Fig. 3)), 2. Carefully expanding the tissue-specific set of eQTLs can improve the rate of misclassification. We now mention this in the discussion secion on page 10, lines 404-411.

   This analysis was more exploratory in nature. If there are individuals with tissue-specific subtypes in the real data, we would expect a larger proportion of individuals to be classified compared to the permuted data in which we attempt to break the genotype-phenotype correlation. In the permuted data, for most of the individuals, the phenotypes

do not have any effect from any of the tissue-specific sets of eQTLs, i.e., no tissue of origin. Hence, tissue-specific subtype posterior probability should be distributed around half for the majority of the individuals. However, no permutation completely unlinks all individuals from the corresponding tissue-specific genetic effect. Hence, even in the permuted case, we expect to observe some individuals classified based on the given threshold of the posterior probability. On the other hand, in original real data, since the two tissues are relevant for the trait, each individual is expected to have tissue-specific genetic effects from at least one of the tissues. In this case, FDR can be defined as the proportion of wrongly classified individuals (tissue 2 instead of tissue 1 or vice versa) among all individuals classified based on the posterior probability threshold. But it is very challenging to estimate FDR for the real data. Thus, we found it very difficult to interpret the permutation results explicitly in terms of FDR.

6. *When looking at the "phenotypic characteristics of individuals with an assigned tissue", it seems to me that you mostly look at heterogeneity compared to the general population. Could you actually compare individuals assigned to tissue A with those assigned to tissue B in a more systematic way and see if there is any phenotype significantly different? To me, that would be very informative to determine somehow a signature for the trait subtypes.*

   Response: We thank the reviewer for the interesting suggestion. We now also compare the two tissue-specific subtype groups between each other to identify quantitative traits heterogeneously distributed between them. We again find that many phenotypes are heterogeneously distributed between the two subtype groups. We now present these results in the supplementary material (Table S23). We discuss the result in the real data analysis section on page 9, lines 371-376.

Reviewer 3:

This study develops a methodology to quantify the tissue-specific genetic contribution to a trait for an individual. The Bayesian methodology uses tissue-specific eQTLs of tissue-specific genes to prioritize tissues. This approach can therefore be used to identify individuals for which the genetic contribution is mediated through the tissue (and therefore potentially identify disease subtypes). The authors then applied the methodology to BMI and WHRAdjBMI in the UK Biobank. There are major concerns with the approach which should be addressed, but I would recommend publication of the paper if these are adequately addressed because the study contains some interesting and novel insights.

Response: We thank the reviewer for the encouraging words.

1. *One of the difficulties I have with the approach is that it does not explicitly consider the scenario in which the genetic contribution to the trait for an individual is mediated through multiple tissues. This scenario may well be the most generic one. The only*

model considered here is one in which the genetic contribution is mediated through a single tissue for an individual, and the extent to which this is realistic or plausible is not clear. The authors recognize this challenge, and, as they point out, in this case the approach would likely distribute the posterior probabilities equally across the tissues.

Response: This is an excellent point. Yes, there will be individuals with their genetic susceptibility mediated through both tissues. Thus, a more general model would be to explicitly accommodate an additional mixture component which will represent the individuals who have genetic contribution from both tissues. In the proposed 2-component model, we anticipate that individuals having genetic contribution from both tissues will have their tissue-specific subtype posterior probability approximately equally distributed across the tissues. We now perform simulation experiments to validate this. For one third of the individuals, the phenotype had an effect from both of the tissue-specific eQTLs. We observe that the tissue-specific subtype posterior probability for this group of individuals is distributed around half. Since, we primarily focus on individuals for whom the genetic susceptibility is mediated through a specific tissue, the simple 2-component model serves the purpose. For simplicity in the current paper, we leave the extension of the model to explicitly accommodate a mixture component which will be assigned to the individuals who have genetic contribution from both tissues as a future work. We provide the simulation results in the supplementary materials (Table S6) and discuss the results in the simulation results section on page 6 (1st paragraph).

2. *Practically, how would one choose the tissues to include in a general application? The authors focused on brain and adipose for BMI, but it's not clear how one would go about selecting tissues to include for a different phenotype.*

   Response: Finucane et al. (Nat Genet, 2018) and Ongen et al. (Nat Genet, 2017) proposed two different statistical approaches to identify the tissues relevant for a phenotype. So for a different phenotype, if previous studies already have not done the analysis, the first step will be to implement these methods to prioritize the relevant tissues. If at least a pair of tissues are identified to be significantly relevant for the phenotype, we can implement eGST based on the prioritized tissues. We now mention this point in the discussion section on page 10, lines 412-418.

3. *Also, the authors used only the top eQTL for each tissue-specific gene. What's the distribution of the number of independent eQTLs for the tissue-specific genes? This will help to clarify/quantify the limitation of the approach.*

   Response: This is an excellent point. Methods such as COJO-GCTA (Yang et al., Nat. Genet., 2012) can be implemented to perform conditional and joint analysis for multiple cis-SNPs to identify independent eQTLs for each tissue-specific expressed gene, while accounting for linkage disequilibrium among the cis-SNPs. We considered top eQTLs mainly for computationally convenient demonstration of our method. It is possible to include the independent eQTLs of each tissue-specific gene. This will likely increase the number of tissue-specific eQTLs, and hence increase the estimate of tissue-specific subtype heritability, and finally the number of classified individuals with a tissue-specific

subtype. We mentioned in 1st submission that including only the top eQTL is one of the main reasons underlying lower estimates of tissue-specific subtype heritability which can be improved by including more eQTLs. Thus, a principled strategy of including more eQTLs will be to perform conditional and joint analysis for multiple cis-SNPs simultaneously accounting for LD structure to detect the independent eQTLs for a tissue-specific gene. However, we anticipate that the main findings will remain similar even after including multiple independent eQTLs in the model, i.e., the genetic susceptibility of a group of individuals is mediated through a specific tissue, and such individuals have distinct phenotypic characteristics which distinguish them from the general population. For simplicity in the current paper, we highlight this idea in the discussion section as a promising strategy of how to include more tissue-specific eQTLs in the model and an important direction for future research work (page 11, lines 435-441).

4. *It's not clear how the uncertainty in the input set of "tissue-specific genes" or the set of tissue-specific eQTLs for such a gene affects the quantification (posterior probability) of the mediating tissue.*

   Response: Some variation in the estimate of tissue-specific subtype posterior probability is expected due to different choices of the set of tissue-specific genes and eQTLs. We considered the top 10% of the tissue-specific expressed genes and the corresponding top eQTLs to implement eGST in UK Biobank. Instead of top 10% genes, we also considered the top 15% of the overexpressed genes in a tissue as the set of tissue-specific expressed genes. Next we ran eGST considering the top eQTL for these selected genes. We observed 73% correlation between the estimate of tissue-specific subtype posterior probability across individuals obtained based on top 10% and 15% tissue-specific expressed genes. If we further increase the percentage of inclusion of tissue-specific genes, it can reduce the tissue-specificity of the selected genes. Thus, how to choose an optimal percentage of tissue-specific genes and corresponding set of eQTLs is a challenging task and requires extensive investigation. However, top 10% expressed genes and the corresponding top eQTLs should always form a core part of the tissue-specific set of genes and eQTLs. Thus, even if we expand the list of tissue-specific expressed genes and corresponding eQTLs, there should be a substantial correlation between the estimates of tissue-specific subtype posterior probability. We now mention this in the discussion section on page 11, lines 442-455.

5. *The authors refer to an R package. Is the source available through github or some other repository? The link should be included.*

   Response: Yes, it is available from CRAN: https://cran.r-project.org/web/packages/eGST/index.html

   We have now included the link at the end of the author summary and at the end of the discussion section.
